# Development of an Electrochemical Immunosensor for Detecting Coagulation Factor Xa and Perspectives in Monitoring Direct Oral Anticoagulant Therapy

**DOI:** 10.1021/acsomega.5c08281

**Published:** 2025-11-07

**Authors:** Mariana Rost Meireles, Julia Konzen Moreira, Giovana Dalpiaz, Muriel Schiling Krohn, Gabriela Victória de Mello Jantzch, Willyan Hasenkamp Carreira

**Affiliations:** † 37906 Universidade do Vale do Rio dos Sinos, UNISINOS, Av. Unisinos, 950., São Leopoldo, RS 93022-000, Brazil; ‡ Biosens Development and Biosensors Industry LTDA (BIOSENS), Av. Theodomiro Porto da Fonseca, 3101, São Leopoldo, RS 93022-715, Brasil; § Universidade Federal do Rio Grande do Sul (UFRGS), Av. Bento Gonçalves, 9500, Porto Alegre, RS 91501-970, Brazil

## Abstract

This study aims to develop a point-of-care (POC) electrochemical immunosensor to monitor the anticoagulant activity of direct oral anticoagulants (DOACs) by detecting and distinguishing between the inactive (FX) and active (FXa) forms of Factor X. DOACs have transformed the management of thromboembolic disorders by selectively inhibiting FXa. Common monitoring methods are either insufficiently sensitive to DOAC levels or not widely accessible. This study demonstrates the development of a graphene-based electrochemical immunosensor modified by gold nanostructures and functionalized with anti-FX antibodies. Electrode modifications were characterized by cyclic voltammetry (CV), differential pulse voltammetry (DPV), and field-emission scanning electron microscopy (FESEM), showing enhanced electron transfer and increased current peaks (*I*_pc_: −241.4 μA, *I*_pa_: 244.6 μA). The sensor distinguished synthetic blood samples with FXa from negative and positive for FX (only 20 μL sample) via DPV peak area analysis (cutoff of 10.65 and 12.42 μA·V). An enzyme-linked immunosorbent assay was performed to verify antigen–antibody interactions, demonstrating less sensitivity to differentiate FX from FXa than DPV. Structural bioinformatics confirmed the loss of a 51-amino acid activation peptide in FXa, reducing molecular volume and likely affecting electrochemical response. In silico docking of seven nonpathogenic F10 variants in DOAC binding regions showed minimal impact on drug binding, supporting sensor applicability across genetic variations. This work demonstrates a sensitive, selective immunosensor for discriminating coagulation factor activation states, supporting point-of-care anticoagulant therapy monitoring.

## Graphical abstract

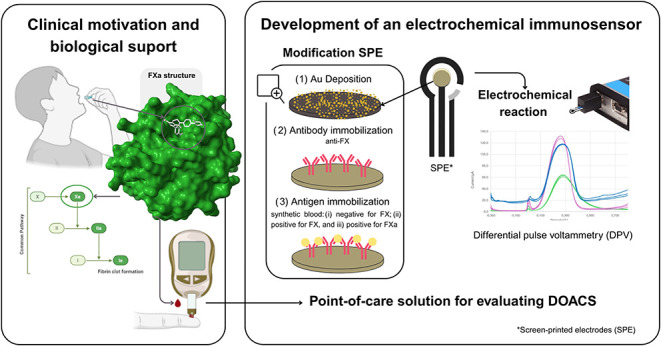

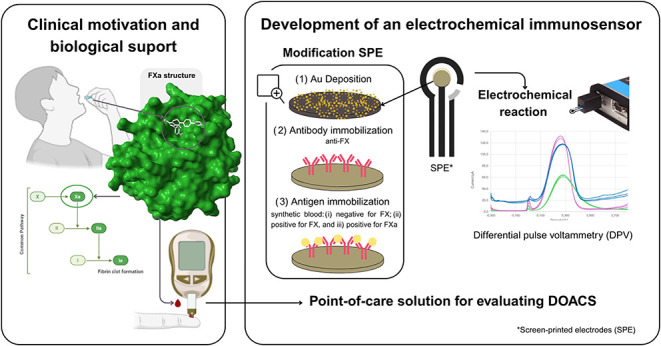

## 1 Introduction

DOAC therapy has revolutionized the clinical approach to the prevention and treatment of thromboembolic events, such as venous thromboembolism (VTE) and stroke prevention in patients with nonvalvular atrial fibrillation (AF). DOACs include direct thrombin inhibitors, such as dabigatran, and direct FXa inhibitors, such as apixaban, rivaroxaban, and edoxaban.^1^ These medications offer significant advantages over vitamin K antagonists (VKAs), such as warfarin, including predictable pharmacokinetics, fewer drug and food interactions, rapid onset of action, and fixed dosing. Although routine laboratory monitoring is not required as frequently as with warfarin, clinical follow-up and occasional assessment of anticoagulant activity remain important. Warfarin, in contrast, demands frequent monitoring and dose adjustments due to its narrow therapeutic window and high interindividual variability. Moreover, its broader mechanism of action, through the inhibition of multiple vitamin K-dependent clotting factors, results in a more generalized anticoagulant effect, increasing the risk of bleeding complications and complicating individualized therapeutic management. In contrast, the more targeted mechanism of DOACs contributes to a better safety profile, fewer dietary restrictions, and improved treatment adherence.^2^ Despite this, there are several clinical situations in which monitoring the anticoagulant effect of DOACs is crucial. These include, for example, suspected overdose, bleeding, and thrombotic events, the need for emergency or elective surgery, progressive renal insufficiency, acute trauma, or in patients on prolonged therapeutic regimens.^3,4^ In these contexts, the accurate quantification of plasma drug levels can provide important support for safe and effective clinical decision-making.

The FX activation process consists of a crucial step for coagulation, turning the FXa into a primary target of several DOACs, including rivaroxaban, apixaban, and edoxaban. These drugs act by directly inhibiting FXa activity, thereby preventing thrombin generation and thrombus formation. FXa is an enzyme from the serine protease family and plays a crucial role in the coagulation. When activated, FXa catalyzes the conversion of prothrombin into thrombin, which subsequently transforms fibrinogen into fibrin, generating a fibrin clot and leading to the formation of a stable blood clot.^5,6^ The coagulation process can be initiated by two distinct paths: (i) tissue injury and release of tissue factor, also known as thromboplastin, in the presence of calcium ions and (ii) contact with negatively charged surfaces (as platelets). Both paths converge at the activation of FX, detaching the central role of this protein.^7^

Currently, laboratory tools available for this purpose are limited. Global coagulation tests, such as prothrombin time (PT), activated partial thromboplastin time (aPTT), and thrombin time (TT), exhibit variable sensitivity and are often inadequate for accurately estimating DOAC activity.^8^ Liquid chromatography coupled with mass spectrometry (LC-MS/MS), although considered the gold standard for quantifying these drugs, requires sophisticated infrastructure, high cost, and prolonged processing time, which limits its routine applicability.^9,10^ Immunological tests, such as FXa-specific enzyme immunoassays, have emerged as promising alternatives; however, their availability is also limited to specialized centers.^11^ In this scenario, the development of POC sensors based on immunological and electrochemical platforms represents an innovative strategy with high clinical potential to enable the decentralized, rapid, and accessible monitoring of DOACs, especially those acting through FXa inhibition.

Electrochemical immunosensors offer several advantages over conventional methods by combining the high specificity of biomolecular recognition with the elevated sensitivity of electrochemical techniques. These devices are already widely explored in various analytical applications and typically employ SPEs modified with nanomaterials, such as carbon nanotubes, metal nanoparticles, and other conductive materials. Such modifications aim to enhance the sensor performance by increasing the active surface area, facilitating electron transfer, and providing lower detection limits. Electrochemical detection is carried out using a portable device that applies electrical stimuli and measures the response generated by the specific interaction between the antigen and the antibody immobilized on the electrode surface.^12^

In addition, electrochemical immunosensors offer rapid response, portability, low manufacturing cost, and miniaturization potential, making them particularly suitable for clinical, screening, or resource-limited settings. Compared to spectroscopic or chromatographic techniques, these sensors do not require complex sample preparation steps or sophisticated laboratory infrastructure, which facilitates their integration into POC devices.^13^

In the context of evaluating FXa inhibition, it is important to understand the details of the interactions between the drugs and the protein, with the aim of establishing binding regions. Establishing this understanding is crucial for better comprehension of the interactions with the bioreceptor and the test’s performance regarding biological variations. In this regard, it is important to consider variations resulting from common and nonpathogenic genetic alterations, such as polymorphisms. These variations must be considered since they often lead to conformational changes in the protein, which do not necessarily cause any disorder but may interfere with DOAC binding.

The presence of missense variants, particularly those located in FXa’s catalytic domain, poses a significant challenge, as even nonpathogenic mutations may alter local physicochemical properties and protein conformation, potentially disrupting drug binding interactions, and may compromise treatment efficacy or safety. In this context, bioinformatics offers powerful tools for investigating such variants, enabling structural modeling, interaction prediction, and functional inference without the immediate need for wet-lab experiments.^14^

To overcome these challenges, this work presents a preliminary development of a graphene-based electrochemical immunosensor aimed at detecting FX and its activation, focusing on its future applicability as a support tool for therapeutic monitoring of patients using DOACs. The proposal aims to contribute effective technological solutions to address current diagnostic gaps, looking for safety and individualization in anticoagulant treatment. Therefore, this study leverages in silico approaches to analyze nonpathogenic *F10* variants, aiming to uncover their potential influence on DOAC–FXa interactions and highlight the importance of integrating computational strategies to consider genetic variants in diagnosis.

## 2 Materials and Methods

The methods involved in sensor procedures and development, such as the procedure of modifying screen-printed electrodes through Au electrodeposition and immobilizing the antibody anti-FX/FXa antibody as the bioreceptor (Section 2.1), characterization of sensors and modifications (Section 2.1), and electrochemical detection of biomolecules FX and FXa as biomarkers (Section 2.2), are summarized in Figure 1 for better comprehension.

**Figure 1:**
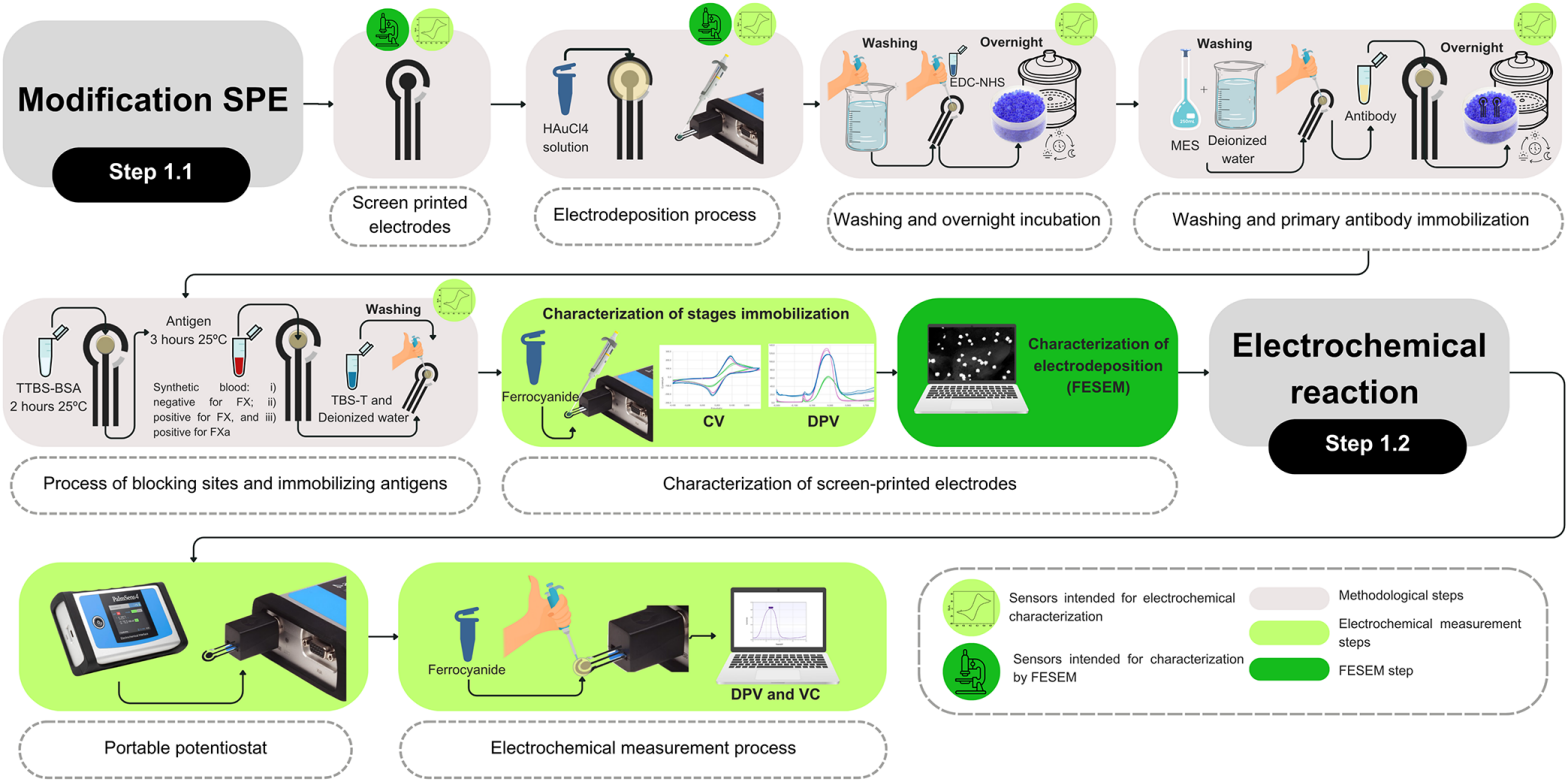
Summary of the immunosensor development steps: The figure shows the steps of modification and electrochemical analysis of screen-printed electrodes (SPE), divided into Steps 1.1 (SPE modification with bioreceptor immobilization) and Step 1.2 (electrochemical detection of the FX and FXa proteins). Step 1.1 includes electrodeposition with HAuCl_4_, procedures for immobilization of the bioreceptor anti-FX/FXa antibody (activation with EDC-NHS, blocking with TTBS-BSA, immobilization of biomarkers), followed by characterization by cyclic voltammetry (CV), differential pulse voltammetry (DPV), and evaluation of electrodeposition by field-emission scanning electron microscopy (FESEM). Step 1.2 corresponds to the electrochemical detection of FX and FXa proteins using CV and DPV, performed with ferrocyanide. The icons indicate that in the respective steps, the sensors were intended for characterization by electrochemical techniques (green graph) or by FESEM (green microscope). The block colors represent methodological steps (gray), electrochemical steps (light green), and FESEM analysis (dark green).

### 2.1 Modifying Screen-Printed Electrodes through Au Electrodeposition and the Immobilization of the Bioreceptor (Anti-FX/FXa Antibody)

Carbon SPEs were fabricated by Biosens LTDA, comprising a three-electrode system with a working electrode (WE), counter electrode (CE), and reference electrode (RE). The WE and CE were printed using a conductive carbon-graphene paste, while the RE consisted of a silver/silver chloride (Ag/AgCl) paste, ensuring a stable reference potential.

The modification of the SPE surface was achieved via gold electrodeposition to enhance the electrochemical properties. This was performed by applying 95 μL of a 5 mM HAuCl_4_ solution onto the electrode surface, followed by applying a constant potential of −0.4 V for 90 s. Subsequently, the electrodes were thoroughly rinsed with deionized water, dried through adsorption to remove any residual reagents, and prepared for further functionalization.

The immobilization process involves (i) incubating 20 μL of EDC-NHS 1-ethyl-3-(3-(dimethylamino)­propyl-*N*-hydroxysuccinimide) compound (1:1) after the completion of electrodeposition to activate the electrode surface for the bioreceptor. This reagent can immobilize biological structures, particularly proteins, due to its amino-terminal portion interacting with the carboxyl groups of amino acids. The incubation was conducted overnight in a controlled humidity environment. (ii) Washing was performed using 30 μL of MES (2-(*N*-morpholino)­ethanesulfonic acid) buffer (25 mM, pH 5.0), followed by rinsing with deionized water to remove possible interference. Subsequently, 20 μL of the capture bioreceptor anti-FX/FXa antibody (1:1000) was immobilized (F8396, Sigma-Aldrich) in a controlled humidity environment overnight. (iii) Then, the surface was blocked with 20 μL of 0.125% TTBS-BSA (Tris-buffered saline with Tween-20-bovine serum albumin) for 2 h at 25 °C. The process was followed by the blood sample (negative and positive for the biomarker) immobilization by pipetting 20 μL and incubating for 3 h at 25 °C. Finally, a wash was performed adding 20 μL of 0.05% TBS-T (Tris-buffered saline with Tween-20) to ensure that only the antigen–antibody complex remained on the sensor surface, followed by rinsing with deionized water to remove possible interference.

#### 2.1.1 Characterization of Screen-Printed Electrodes

The electrochemical behavior of both unmodified and modified sensors functionalized by gold electrodeposition and anti-FX/FXa antibody immobilization was characterized using three techniques: CV and DPV. Proper controls were performed using DPV to confirm sensor specificity, including basal sensors, sensors with only the capture antibody, sensors with only the antigen, and the complete antigen–antibody complex formation; the corresponding data are presented in Figure S1 (Supporting Information). All measurements were performed using a portable potentiostat (PalmSens 4), with data acquisition and analysis conducted via PSTrace 5.11 software. CV was employed to investigate the redox activity and assess changes in the electrochemical profile during each modification step, which are indicative of surface modification and biomolecular interactions. DPV was selected for its high sensitivity in detecting electroactive species, enabling precise evaluation of the sensor’s analytical performance.^15^

The CV was performed in 10 cycles with an *E* begin of 0.0 V, an *E* step of 0.01 V, and a scan rate of 0.05 V/s. The fifth cycle of each measurement was selected for comparisons and evaluations because the current–potential profile stabilized after the initial scanning cycles according to the literature reports.^16–18^ Initial cycles (1–3) showed small variations consistent with surface reorganization and equilibration following electrodeposition and functionalization; thereafter, for cycles 4–6, the voltammograms reached a reproducible shape and peak current values, while later cycles (7 and 10) may introduce minor alterations in surface properties due to restructuring or adsorption. Although the cycles demonstrate stability in our tests, the fifth cycle was chosen as an equilibration point. Representative stabilization data are provided in the Supporting Information (Figure S2). Choosing a stabilized cycle reduces the influence of transient effects and provides a reproducible basis for comparing the electrode modification steps.

The DPV setting parameters include *E* begin, *E* end, and *E* step of −0.5, 0.5, and 0.01, respectively, *E* pulse of 0.05, *t* pulse of 0.1s, and 0.02 V/s for scan rate. The measurements were performed using 20 μL of Fe­(CN)_6_^3–^/^4–^. All conditions were tested in triplicate using independent sensors to prevent damage to the SPE surface caused by Fe­(CN)_6_^3–^/^4–^, avoid sensor-to-sensor bias, and support the robustness of the platform for future sensing applications. For data analysis, the peak area (μA·V), width (V), and height (μA) were extracted from DPV. From CV, the cathodic (*I*_pc_) and anodic (*I*_pa_) peak currents were obtained, along with their corresponding peak potentials (*E*_pc_ and *E*_pa_). Subsequently, the peak potential difference (Δ*E*_p_ = |*E*_pa_ – *E*_pc_|) and the peak symmetry ratio (*I*_pa_/*I*_pc_) were calculated. These parameters are commonly used to assess the reversibility of the redox process and the electron transfer kinetics of the electrochemical system.

The morphology of the gold nanostructures formed during the Au electrodeposition process was examined by FESEM. Imaging was performed using an Inspect F50 (FEI) microscope operating at an accelerating voltage of 20 kV, enabling a high-resolution visualization of the electrodeposited gold features. To correlate surface morphology with electrochemical performance, FESEM images were compared with CV and DPV data to evaluate the influence of nanostructure distribution, grain size, and surface area on the sensor response.

### 2.2 Electrochemical Reaction with Biomarker

Samples definition: Aiming to evaluate the sensor performance in biomarker FX and FXa detection, three distinct types of samples were considered: (i) synthetic blood for hematologic tests without coagulation proteins - negative for FX, (ii) synthetic blood for coagulation test - positive for FX, and (iii) synthetic blood for coagulation test containing FX further activated with thromboplastin to generate FXa. These control samples were obtained from Controllab in a lyophilized form and reconstituted with 100 μL of ultrapure water prior to use. Positive and negative samples contain interferents typically found in real blood, such as erythrocytes, leukocytes, and platelets, which may affect the electrochemical response but must be considered in point-of-care device development. The negative sample does not present the target biomarker (FX/FXa); although it contains cellular components, it is not intended for coagulation testing and therefore lacks FX, a key protein in this process. To obtain the positive sample containing the activated biomarker (FXa), an activation procedure commonly used in coagulation assays was applied, consisting of the addition of thromboplastin, a coagulation reagent composed of tissue factor and phospholipids that triggers the extrinsic coagulation pathway and leads to FX activation. This activation procedure was applied to differentiate between samples containing inactive FX and active FXa, which is critical to assess the immunosensor’s sensitivity and specificity toward these biomarkers.

To perform these tests, 20% μL of each control sample was pipetted onto the sensors, and the devices were incubated for 3 %h at 25% °C. Following incubation, 30% μL of ferricyanide in KCl (K4­[Fe­(CN)­6]) were added to the detection area. Biomarker (FX and FXa) detection was performed using DPV, applying the same parameters previously established during sensor characterization and immobilization validation. For data analysis, the peak area (μA·V), width (V), and height (μA) were extracted from DPV.

Based on the integration of the DPV curve expressed in microamperes μA, it was possible to determine the cutoff values that differentiate between sample groups. The first cutoff corresponds to the threshold that separates the electrochemical responses of negative samples for Factor X from those positive for FX. The second cutoff distinguishes samples positive for FX from those containing activated Factor X. These cutoff values were calculated using the mean and standard deviation of the current responses from negative samples (for the first cutoff) and from FX-positive samples (for the second cutoff), according to the following equation (Cutoff = *x̅* + *z* · σ) where *x̅* represents the mean current of the analyzed samples, σ is the standard deviation, and *z* is the confidence factor (for example, *z* = 2 corresponds to a 95% confidence level).^19^

### 2.3 Validation of Antibody Affinity to FX and FXa Samples through ELISA and Conformational Investigation

An indirect sandwich ELISA was performed to evaluate the binding specificity of the anti-FX/FXa antibody to the biomarkers FX and FXa in synthetic control blood samples, assess potential cross-reactivity, and establish a detection profile. The antibody used in this research was the monoclonal anti-FX antibody produced in mouse (F8396, Sigma-Aldrich) coated in a 96 wells plate (2% μg/mL in phosphate-buffered saline (PBS); 100% μL/well) and incubated overnight at 4% °C. Wells were washed three times with TBS-T (20 mM Tris-HCl, 150 mM NaCl, 0.05% Tween-20, pH 7.4). Nonspecific binding sites were blocked with 1% bovine serum albumin (BSA) in TBS-T (200 %μL/well) for 1.5 %h at 37% °C, followed by five additional washes with TBS-T. Then the control samples (200% μL/well) were added in 7-point serial dilutions (1/2, 1/4, 1/8, 1/16, 1/32, 1/64, 1/128) to determine the limit of detection (LOD) and incubated at 37% °C for 1%h. The samples included (I) synthetic whole blood deficient in coagulation factors, (II) synthetic control whole blood with FX, and (III) synthetic whole blood with FX and activated by thromboplastin. The limit of detection (LOD) was estimated based on the optical density (OD) of the negative control samples. The LOD threshold was defined as the mean OD of the negative controls plus three times their standard deviation, allowing identification of the lowest dilution at which FX or FXa could be reliably distinguished from the background.

After washing (5× TBS-T), anti-FXa antibody (100% μL/well) was added, and the plates were incubated for 1% h at 37% °C. Plates were washed again (5×), followed by the addition of horseradish peroxidase (HRP)-conjugated secondary antibody (100 %μL/well) and incubation for 1% h at 37% °C. Another five washes with TBS-T were performed. The detection was performed by adding 100% μL of tetramethylbenzidine (TMB) substrate to each well, and the reaction was developed for 10 min in the dark at room temperature. The reaction was stopped by adding 50% μL of 1% M H_2_SO_4_ per well, and absorbance was measured at 450% nm using a microplate reader.

Complemental structural bioinformatic analyses were performed to verify structural modifications caused by the activation process in the FX protein conformation. The Protein Data Bank (PDB) identity (ID) 1C5M represents the FXa, post activation peptide cleavage, and a modeling approach was conducted using I-Iterative Threading ASSEmbly Refinement (TASSER) to generate inactive FX. The visual inspection of structures was made through Chimera interface to modeler.

### 2.4 Comprehension of DOAC Binding Interaction Sites in FXa and Nonpathogenic Variants’ Effect on Interaction

A multistep in silico approach was employed to investigate the binding sites of DOACs with FXa and how nonpathogenic missense variants of the F10 gene might influence these interactions. First, 45 asymptomatic variants located in the serine protease domain (SP) were selected from the European Association for Hemophilia and Allied Disorders (EAHAD) database. Crystallographic structures of FXa complexed with rivaroxaban, letaxaban, and apixaban DOACs (PDB: 2W26, 3KL6, and 2P16, respectively) were analyzed using ChimeraX and LigPlot+ to identify key binding residues, as shown in Figure 10.

Seven of the selected variants were found to colocalize with drug interaction sites. These variants were assessed using five variant effect prediction algorithms (SIFT,^20^ PolyPhen-2,^21^ PROVEAN,^22^ HOPE,^23^ and SNAP2 ^24^) to infer potential functional impacts. A homology modeling approach Phyre-2 ^25^ (one-to-one threading job mode) was applied to generate models for each variant and wild type (WT). The model’s qualities were validated via QMeanDisco and SAVES tools. Finally, molecular docking analyses were performed using AutoDock Vina to estimate the impact of each variant on DOAC binding, compared against the WT. Redocking was used to validate docking parameters, binding energies, and root mean square deviation (RMSD) values. Those data were analyzed and compared among variants and the wild structure to infer potential alterations in drug affinity.

## 3 Results and Discussion

### 3.1 Characterization of Screen-Printed Electrode Modifications

The modifications on the SPE (including gold electrodeposition, EDC-NHS functionalization, and capture antibody (cAb) immobilization) were reflected in the electrochemical responses obtained by CV and DPV. The analysis evidenced the reproducibility of the sensors and the distinguishable patterns related to each modification step. Figure 2 depicts the voltammograms for each condition, and raw data were compiled into spreadsheets and are provided as Supporting Information (Table S1). All electrochemical measurements were performed in triplicate (*n* = 3), and the results are expressed as mean ± standard deviation, confirming reproducibility of the sensor fabrication and functionalization process.

**Figure 2:**
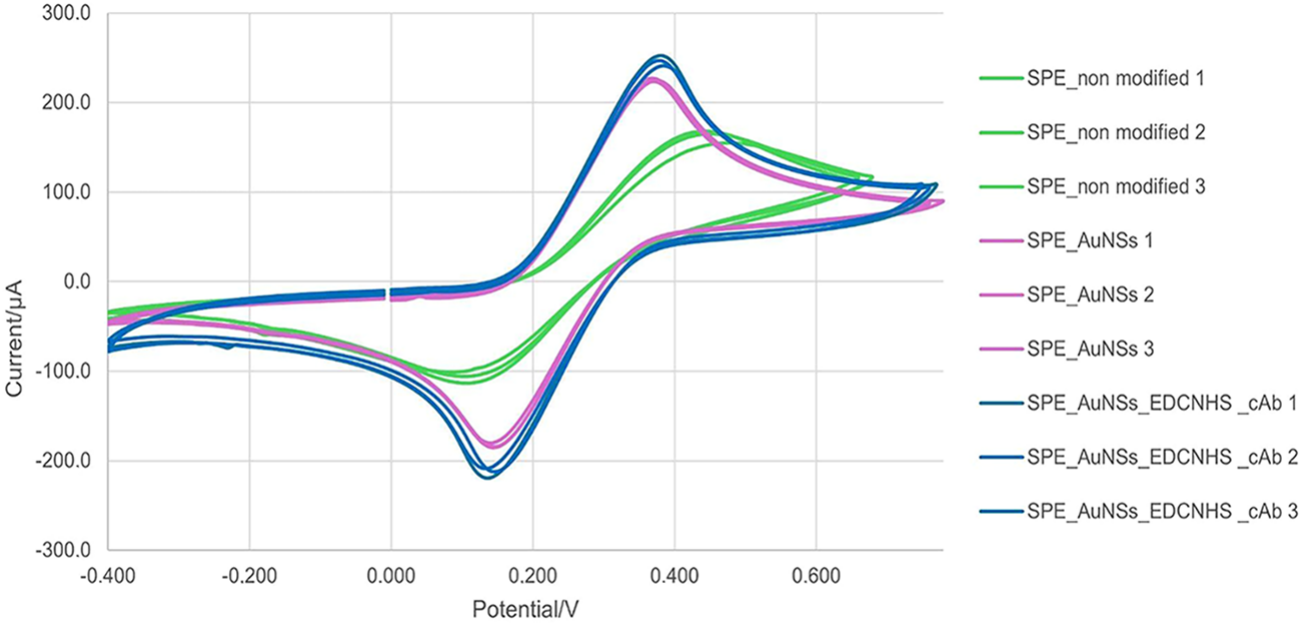
Characterization of SPE modification processes and their effects in cyclic voltammetry. Cyclic voltammograms were recorded for the sensor under different conditions. The curves correspond to green, unmodified sensor (SPE_nonmodified); pink, sensor after gold electrodeposition that generates gold nanostructures (SPE_AuNSs); blue, sensor after functionalizing with EDC-NHS and immobilization of capture antibody (SPE_AuNSs_EDCNHS_cAb). Variations in anodic and cathodic peak currents indicate changes in the electrochemical response, according to each condition.

Considering the characterization depicted in Figure 2, data revealed that in the SPE_unmodified condition, an *I*_pc_ of −109.46 μA ± 7.98 and an *I*_pa_ of 168.64 μA ± 5.60 were detected, with a potential difference between peaks (Δ*E*_p_) of 0.32 V and a peak symmetry ratio (*I*_pa_/*I*_pc_) of 1.55. In the SPE_AuNS condition, a significant increase in current peaks was observed, with an *I*_pc_ of −224.00 μA ± 2.58 and an *I*_pa_ of 238.62 μA ± 4.20. There was also a reduction in Δ*E*_p_ to 0.22 V and an improvement in peak symmetry (*I*_pa_/*I*_pc_ = 1.06). In the SPE_AuNSs_EDC/NHS_cAb condition, the *I*_pc_ reached −241.40 μA and the *I*_pa_ reached 244.59 μA ± 6.68, with a Δ*E*_p_ of 0.24 V and a symmetry ratio even closer to the ideal (1.01).

These results indicate that the electrode modification by electrodeposition of gold nanostructures (AuNSs) significantly contributes to the system’s sensitivity, evidenced by the increase in current peaks, related to a higher electron transfer rate at the electrode/solution interface, the improvement in peak symmetry (*I*_pa_/*I*_pc_ values closer to 1, indicating more reversible and challenging redox behavior), and the reduction in Δ*E*_p_. The lower the Δ*E*_p_, the more reversible the redox process, reflecting more efficient electrode kinetics and a lower need for an overpotential for reactions to occur. Furthermore, sensitivity was enhanced by the immobilization of biological bioreceptors, possibly due to the increased specificity of the antibodies, which facilitated enhanced molecular recognition and selectivity, thereby favoring analyte accumulation at the interface. The presence of bioreceptors may also have promoted more efficient organization of the functionalized surface, facilitating electron transfer and resulting in more intense electrochemical signals and results.^26^

The data obtained by DPV, as depicted in Figure 3, show a progressive increase in peak area, height, and width as the electrode surface is modified. In the SPE_unmodified conditions, the peak area was 13.80 μA·V ± 0.81, the width was 0.21 V ± 0.01, and the height was 61.85 μA ± 5.76. With the modification using SPE_AuNSs, an increase in peak area to 17.37 μA·V ± 0.54 and peak height to 97.43 μA ± 3.02 was observed, along with a slight reduction in peak width (0.18 V ± 0.01), indicating signal amplification due to the enhanced conductivity and surface area provided by the AuNSs. After functionalization with EDC/NHS and antibody immobilization (SPE_AuNSs_EDC/NHS_cAb), the peak area further increased to 19.59 μA·V ± 0.92 and the height to 100.19 μA ± 5.46, maintaining a width of 0.20 V ± 0.01. These results confirm both the successful modification of the electroactive surface and the effective immobilization of bioreceptors, which promote a favorable interface for analytical recognition. Replicates (*n* = 3) showed consistent behavior across devices, with relative standard deviations (RSD) for peak current values of 9.3 % for SPE_unmodified, 3.1%% for SPE_AuNSs, and 5.5 %% for SPE_AuNSs_EDC/NHS_cAb, supporting the reproducibility of the fabrication steps.

**Figure 3:**
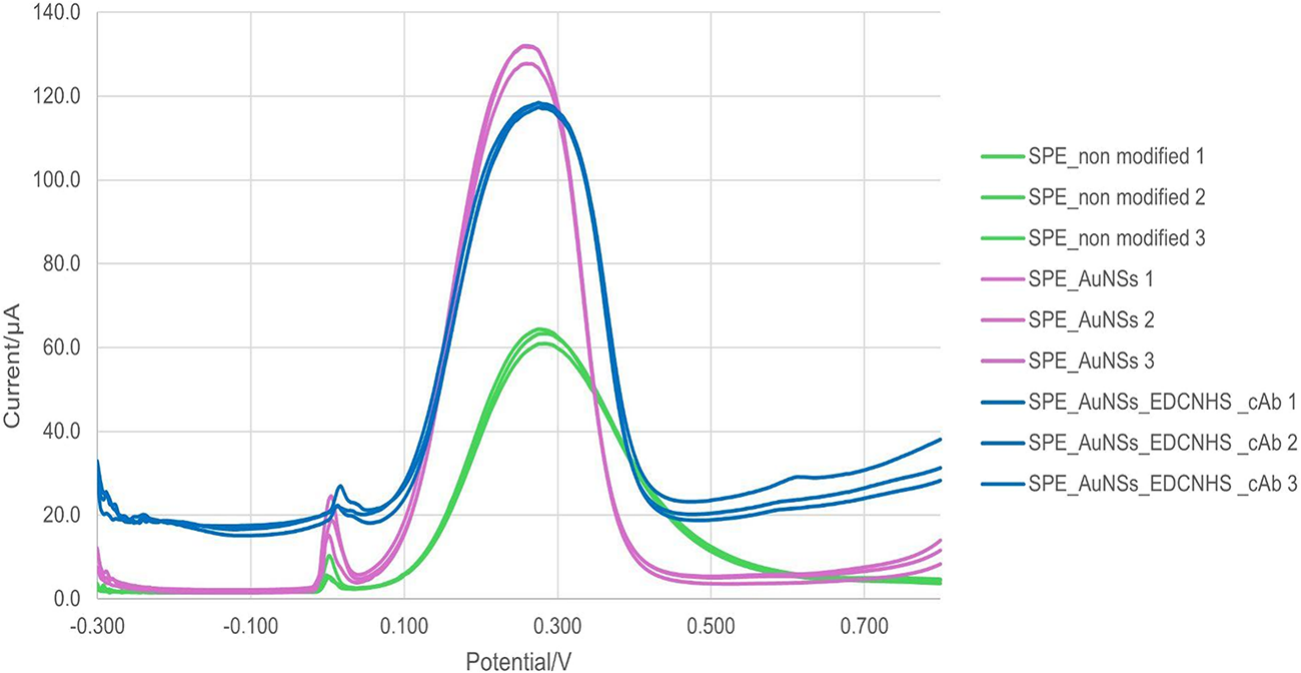
Characterization of SPE modification processes and their effects in differential pulse voltammetry. DPV was recorded for the sensor under different conditions. The curves correspond to green, unmodified sensor (SPE); pink, sensor after gold electrodeposition that generates gold nanostructures (SPE_AuNSs); blue, sensor after functionalizing with EDC-NHS and immobilization of capture antibody (SPE_AuNSs_EDCNHS_cAb).

The gold electrodeposition effect in surface modification was also confirmed in the FESEM, which allowed for the comprehension of the distribution, morphology, and grain size of the AuNSs generated on the sensor surface. The AuNSs measured approximately 0.4% μm on average, ranging from 0.1 to 0.7%μm, and exhibited a spiky, nonsmooth morphology rather than flat surfaces. Figure 4 depicts the differences in the sensor surface before and after AuNS formation, considering FESEM analysis.

**Figure 4:**
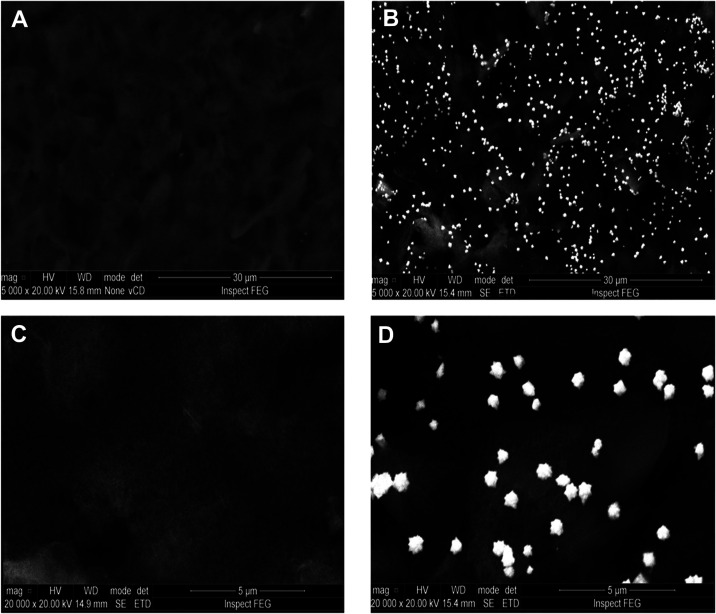
Images obtained by field-emission scanning electron microscopy. In part A, a nonmodified WE from the SPE with a magnification of 5000×; in part B, the same condition can be observed with a magnification of 20,000×. In parts C and D, WE from SPE after the gold electrodeposition procedure, forming gold nanostructures (AuNSs) with a magnification of 5000× and 20,000×, respectively.

The FESEM images (Figure 4) revealed the SPE surface roughness and heterogeneity, which increase the effective surface area and create physical sites for coupling molecules. These characteristics are consistent with the sensors′ fabrication process using a graphene–carbon paste during the screen-printing process. Moreover, the heterogeneity in the surface contributes to enhanced electron transfer rates by exposing more electroactive regions, leading to the amplification of the electrochemical signal.^27,28^

Figure 4 also highlights the formation and distribution of AuNSs on the sensor surface. A combined analysis of the FESEM images with the CV and DPV graphs (Figures 2 and[Fig fig3]) suggests that the presence of gold nanostructures generated by the electrodeposition process enhances the sensitivity of the SPE. The spiky morphology of the AuNSs provides a high density of sharp tips, which enhances electron transfer rates by exposing more electroactive regions. This is evidenced by CV measurements, where the redox peaks of Fe­(CN)_6_ become more pronounced and exhibit greater amplitude, increasing the peak-to-peak separation (Δpeak). The nanoscale features of AuNSs facilitate a faster charge transfer and provide a larger number of active sites for immobilization.

The incorporation of gold in the sensor, in this case in the form of AuNSs, plays a key role in this enhancement due to its excellent electrical conductivity and ability to facilitate fast charge transfer.^29,30^ In addition, AuNSs serve as highly efficient platforms for immobilizing thiol- or amine-modified biomolecules, thereby improving both the stability and orientation of capture probes on the electrode surface.^31^ Consequently, the specific size and spiky morphology of the AuNSs observed via FESEM provide a direct explanation for the improved electrochemical responses observed in DPV, with more defined signals and lower detection limits. Moreover, the morphology of the AuNSs offers more effective anchoring points for the anti-FX/FXa bioreceptor immobilization efficiency, probe orientation, and overall stability of the biosensor.

Considering the EDC-NHS addition and antibody immobilization process (SPE_AuNS_EDCNHS_cAb), the observed increase in peak currents suggests the presence of activated ester groups from EDC-NHS, which enhance the surface polarity and promote electrostatic interactions with the negatively charged redox probe. This compound also plays a role as a functionalizing agent, providing groups for covalent binding of the antibody. The washing steps that follow antibody immobilization can potentially contribute to maintaining an ordered protein layer that does not significantly hinder the electron transfer. This explains why the elevated current response is preserved after antibody addition, contrary to the typical blocking effect expected from the protein layers.

Those observations were also supported by DPV analysis, where the baseline SPE presented a broader and flatter peak reflecting a lower current intensity and a wider potential range, indicating a slower and more dispersed electron transfer. After the gold electrodeposition procedure, the peak became sharper and more intense, indicating an improvement in the kinetics. Following the activation with EDC-NHS and antibody immobilization, the peak onset shifted upward (indicating a higher baseline current), while the peak height slightly decreased and the peak width increased again. This behavior suggests a combination of increased capacitive background and mild resistance to charge transfer, likely caused by the presence of functional and biological layers on the electrode surface.

This discrepancy can be attributed to inherent differences between the two electrochemical techniques. CV involves a continuous potential sweep and captures both faradaic and capacitive currents, including contributions from charge storage and interfacial phenomena, which can amplify the observed current response. In contrast, the DPV operates by measuring the current immediately before and after each potential pulse and computing the differential signal. This approach effectively eliminates nonfaradaic background, minimizing capacitive and charging currents, thereby providing a more selective measure of direct electron transfer kinetics.

In general, both measurements confirmed the success of the sensor modification steps and revealed the characteristics of each stage, providing a potential basis for future validation. The increase in the peak currents in CV in SPE_AuNSs_EDCNHS_cAb (EDC-NHS activation and antibody immobilization) was not similarly reflected in DPV measurements. This discrepancy can be attributed to inherent differences between the two electrochemical techniques.^32^ CV involves a continuous potential sweep and captures both faradaic and capacitive currents, including contributions from charge storage and interfacial phenomena, which can amplify the observed current response.^33^ In contrast, DPV operates by measuring the current immediately before and after each potential pulse and computing the differential signal. This approach effectively eliminates nonfaradaic background, minimizing capacitive and charging currents, thereby providing a more selective measure of direct electron transfer kinetics.^34^

These variations in the electrochemical measurements employed explain the differences observed in the characterization of the immobilization process. The presence of EDC-NHS can induce capacitive or pseudofaradaic effects in CV, while in DPV, the resistance introduced by antibody addition may play a more prominent role, leading to a broader and lower DPV peak. It is emphasized that while CV highlights overall interfacial changes including capacitive behavior, DPV offers a more accurate assessment of the redox probe’s electron transfer dynamics.

### 3.2 Electrochemical Reaction with Biomarkers

This step was essential to assessing whether our biosensor could maintain specificity and signal discrimination in a complex biological matrix. Additionally, since DOACs act specifically on FXa, it is crucial to differentiate between activation states.

When the negative sample for FX is compared with the positive sample, a distinct electrochemical behavior can be observed, as shown in Figure 5. The condition SPE_AuNSs_EDC/NHS_cAb_negative sample FX showed a peak area of 8.91 μA·V ± 0.87, a width of 0.16 V ± 0.01, and a peak height of 54.49 μA ± 5.93. In contrast, the SPE_AuNSs_EDC/NHS_cAb_positive sample FX condition presented a peak area of 10.82 μA·V ± 0.80, a width of 0.19 V ± 0.01, and a height of 56.87 μA ± 4.51. Negative samples exhibited a smaller peak area and narrower peak width compared to positive samples, indicating a less pronounced electrochemical response in the absence of the target. Based on these values, a cutoff for the peak area was established at 10.65 μA·V between these two conditions.

**Figure 5:**
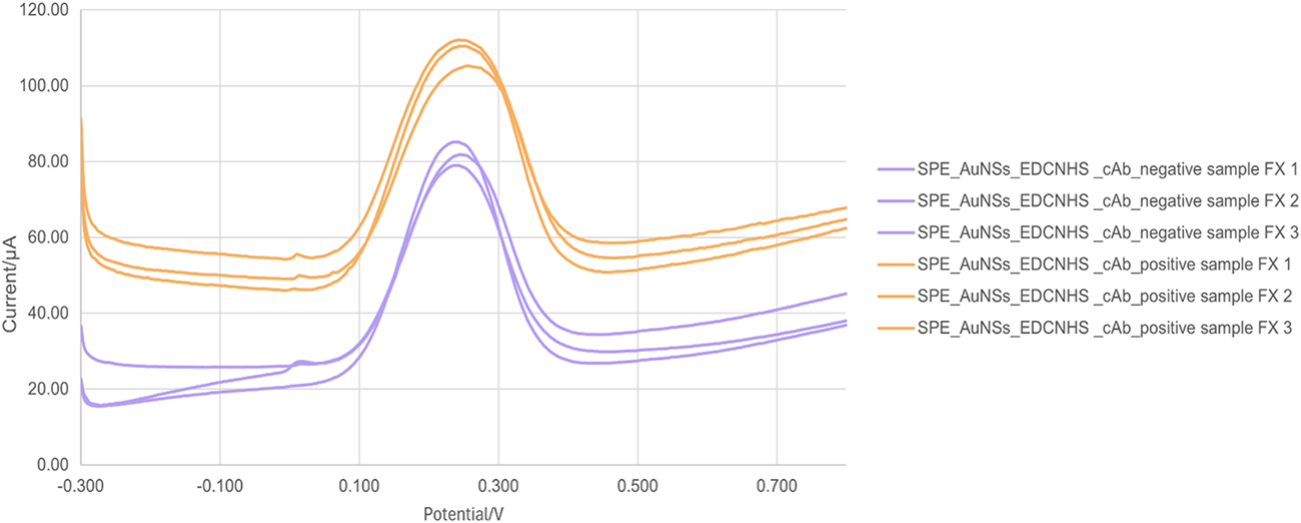
Electrochemical differentiation between negative and positive blood samples for Factor X using differential pulse voltammetry (DPV). DPV measurements were recorded for the sensor under different conditions. The curves correspond to orange, negative sample for FX (SPE_AuNSs_EDC/NHS_cAb_negative sample FX); purple, positive sample for FX (SPE_AuNSs_EDC/NHS_cAb_positive sample FX).

Following the successful differentiation between blood samples containing FX and negative controls, we proposed a new experimental approach focused on the specific action of DOACs, which target the activated form of FXa. This approach involved inducing coagulation in whole blood samples to promote the activation of FX and subsequently performing a comparative analysis between activated and nonactivated samples. The goal was to simulate a more physiologically relevant environment and assess the sensor’s ability to distinguish FXa presence under conditions that better reflect the clinical context of anticoagulant therapy. Figure 6 shows the results of this test.

**Figure 6:**
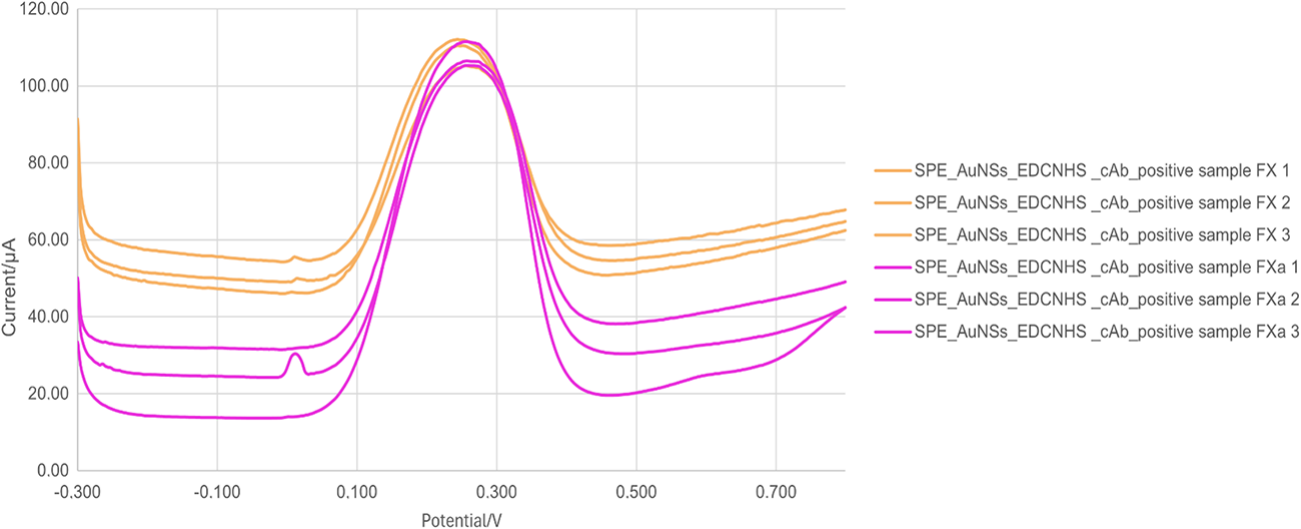
Electrochemical differentiation between positive blood samples for Factor X and Factor X activated by using differential pulse voltammetry (DPV). DPV measurements were recorded for the sensor under different conditions. The curves correspond to orange, negative sample for FX (SPE_AuNSs_EDC/NHS_cAb_negative sample FX); pink, positive sample for FXa activated (SPE_AuNSs_EDC/NHS_cAb_positive sample FXa).

Based on the data obtained, clear differences can be observed between the two positive samples and in comparison to the negative sample. For the SPE_AuNSs_EDC/NHS_cAb_positive sample FXa condition, the peak area was 16.15 μA·V ± 2.40, the width was 0.20 V ± 0.01, and the peak height was 81.07 μA ± 12.04. These results demonstrate that the peak area, height, and width are higher in samples containing FXa compared to the other conditions evaluated due to the amplification of the electrical signal generated by the antigen–antibody binding. Based on the positive sample data, it was also possible to establish a second cutoff for the peak area, set at 12.42 μA·V, to differentiate samples containing FX from those with FXa, as illustrated in the box plot of Figure 7.

**Figure 7:**
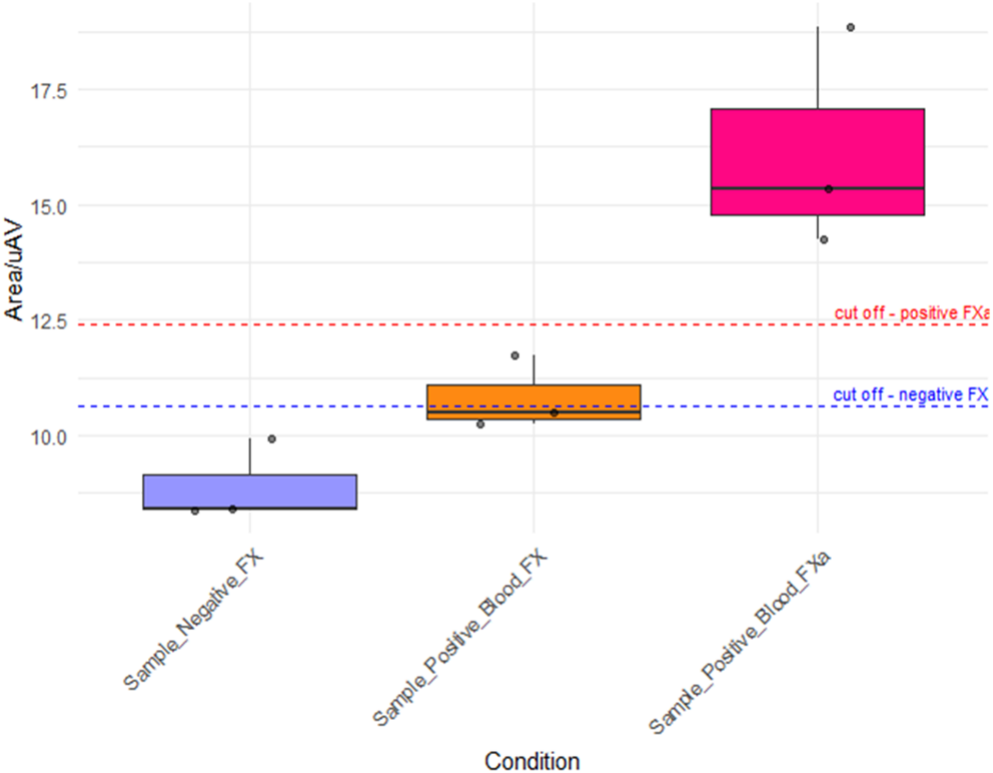
Data distribution among conditions and cutoff considering Area/μA·V. Box plot illustrating the electrochemical response under three conditions: FX-negative samples (lilac), FX-positive samples (yellow), and FXa-positive samples (pink). The established cutoff values used to differentiate FXa from FX-negative (10.65 μA·V) and FX-positive (12.42 μA·V) are indicated by dashed lines. These thresholds support the sensor’s ability to discriminate between different activation states of FX in blood samples. All values were obtained from three independent replicates per condition (*n* = 3), with standard deviations reported in the text. The reproducibility observed across replicates reinforces the robustness of the cutoff determination.

In samples containing inactive FX, compared to negative controls, the peak onset exhibited an upward shift, indicating an increased baseline current. This observation suggests that the presence of inactive FX induces modifications at the electrode interface, potentially due to protein adsorption or alterations in the surface properties. Following activation of FXa, this baseline shift was no longer detected. Additionally, a significant difference in the peak area (expressed in μA·V) was observed between the FXa and other conditions.

Although these differences are clinically relevant and since the recognition of FXa is the primary target of DOACs, further investigation was undertaken to understand the factors influencing the observed results. Consequently, the next step involved performing a classical immunoassay using the same samples and antibody to evaluate their individual analytical performance, complemented by a structural bioinformatics analysis to provide deeper insight into the observed phenomena.

### 3.3 Validation of Antibody Affinity to FX and FXa through ELISA Technique

The capture antibody targets an epitope on the light chain of FX, a region structurally shared by both FX and its activated form, FXa, which is also associated with inhibitory action on the factor. The ELISA assay provides an evaluation of the antibody’s performance in detecting FX and FXa in synthetic whole blood samples. The ELISA results showed no differences in antibody interaction with FX or FXa, making it impossible to distinguish the performance between these two forms based on this assay. Figure 8 shows the optical density (OD) values obtained from the ELISA for the different samples, starting at a 1:2 dilution to 1:128.

**Figure 8:**
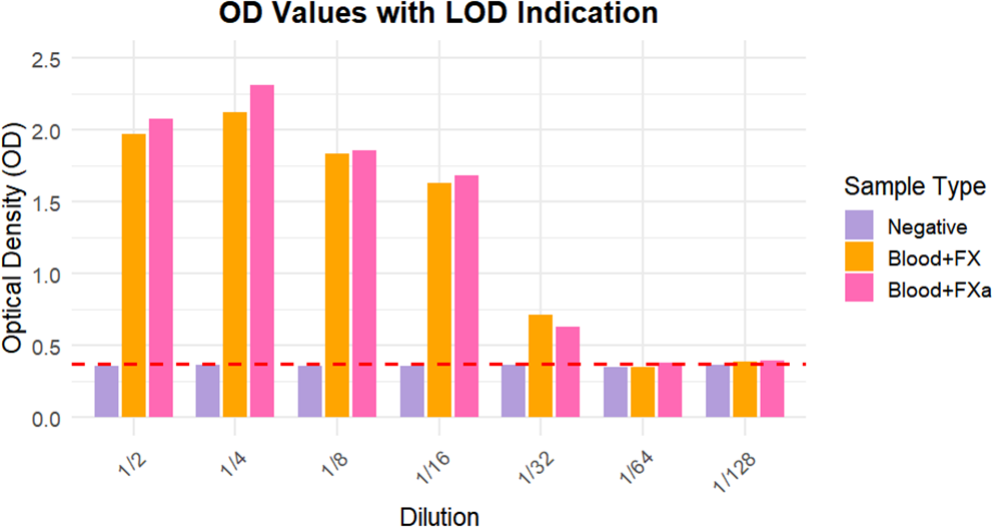
Data distribution of ELISA results: Optical density (OD) among the sample types and dilutions. The negative, synthetic blood with FX, and synthetic blood with FXa are represented by lila, salmon, yellow, and pink bars, respectively. The graph expresses the anti-FX binding with both states of FX: inactive and active. The results demonstrate that positive samples could be distinguished from the negative control down to a 1:32 dilution, establishing the antibody’s limit of detection (LOD) under physiologically relevant conditions.

The positive control samples used in this ELISA correspond to standard coagulation control blood containing Factor X at physiological concentrations (approximately 8–10 μg/mL). This setup allows evaluation of the antibody’s capability to detect FX under conditions relevant to human blood. The observed OD values across serial dilutions demonstrated that positive samples could be distinguished from the negative control down to a 1:32 dilution, indicating the antibody’s ability to discriminate FX presence under physiologically relevant conditions. While this LOD derived from ELISA is not directly equivalent to the detection limit of our electrochemical sensor, it validates the anti-FX/FXa antibody as a reliable bioreceptor. Importantly, we emphasize that the electrochemical sensor itself was not tested in serial dilutions, as its purpose is not to quantify FX over a wide range of concentrations but rather to detect its presence at physiological levels in human blood without the need for sample dilution. This highlights the feasibility of the sensor to operate under clinically relevant conditions, supporting its application in rapid and portable point-of-care testing.

The test results revealed a relevant finding: the test using DPV measurement on the electrochemical sensor demonstrated greater sensitivity in distinguishing between FX with and without the activation process. These results may be related to the conformational change caused by the cleavage of the activation peptide, which reduces the protein’s size and may decrease the resistance associated with the protein layer. The structural bioinformatic analyses performed, including FX modeling, were employed in an attempt to understand these patterns.

There are significant structural changes induced by activation that can enable discrimination between activated FXa and its zymogen form, FX. The cleavage and consequent loss of the activation peptide not only reduce the overall molecular volume of the target protein (Figure 9) but also modify its surface properties. Although these structural alterations do not appear to affect antibody binding, as evidenced by ELISA data and manufacturer information, they seem to be reflected in changes in the electrochemical profile observed in DPV. This may be attributed to the removal of approximately 51 amino acids during activation, which is likely associated with a corresponding decrease in resistance. Additionally, activation disrupts certain intramolecular interactions, leading to a distinct folding and different exposures of residues. Those facts could contribute directly to modify the surface physicochemical properties due to exposure of new amino acid side chains (Figure 10).

**Figure 9:**
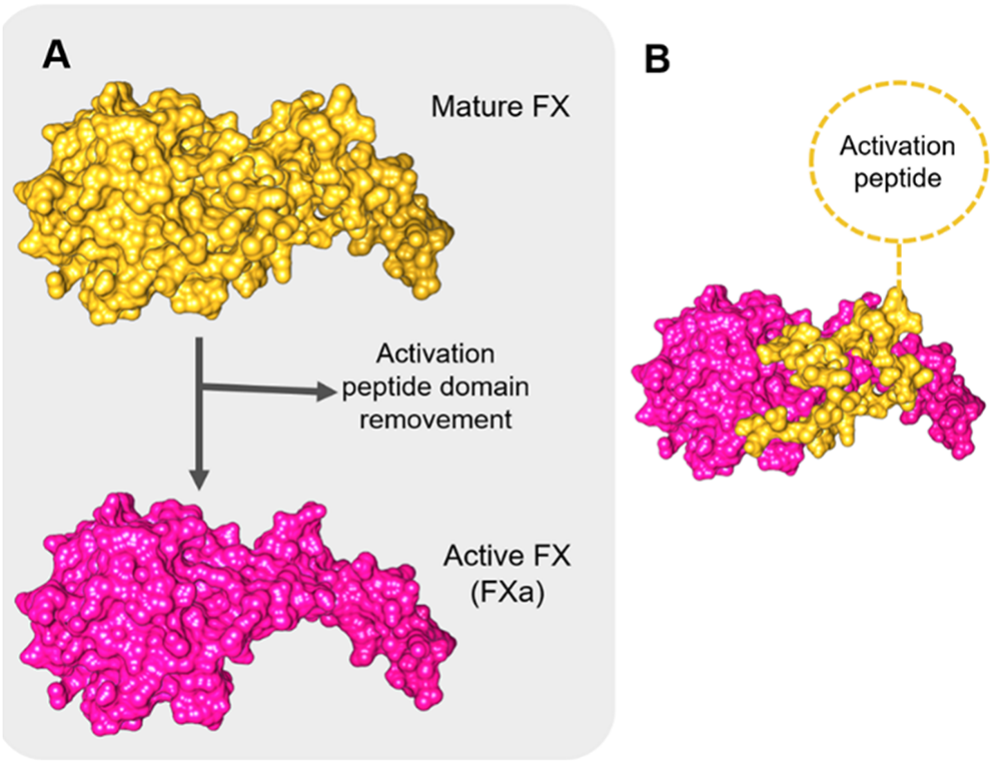
Conformational changes induced by FX activation: In part (A), cleavage of the activation peptide (AP) by the tenase complex produces FXa, a protein that is 51 amino acids shorter. The loss of the AP affects not only the overall size of the protein but also its folding. Additionally, regions previously buried become exposed and are available for interactions. The FX and FXa are represented in yellow and pink, respectively. In part (B), FX is depicted with AP detached in yellow, while the structure that remains after the activation process is shown in pink.

**Figure 10:**
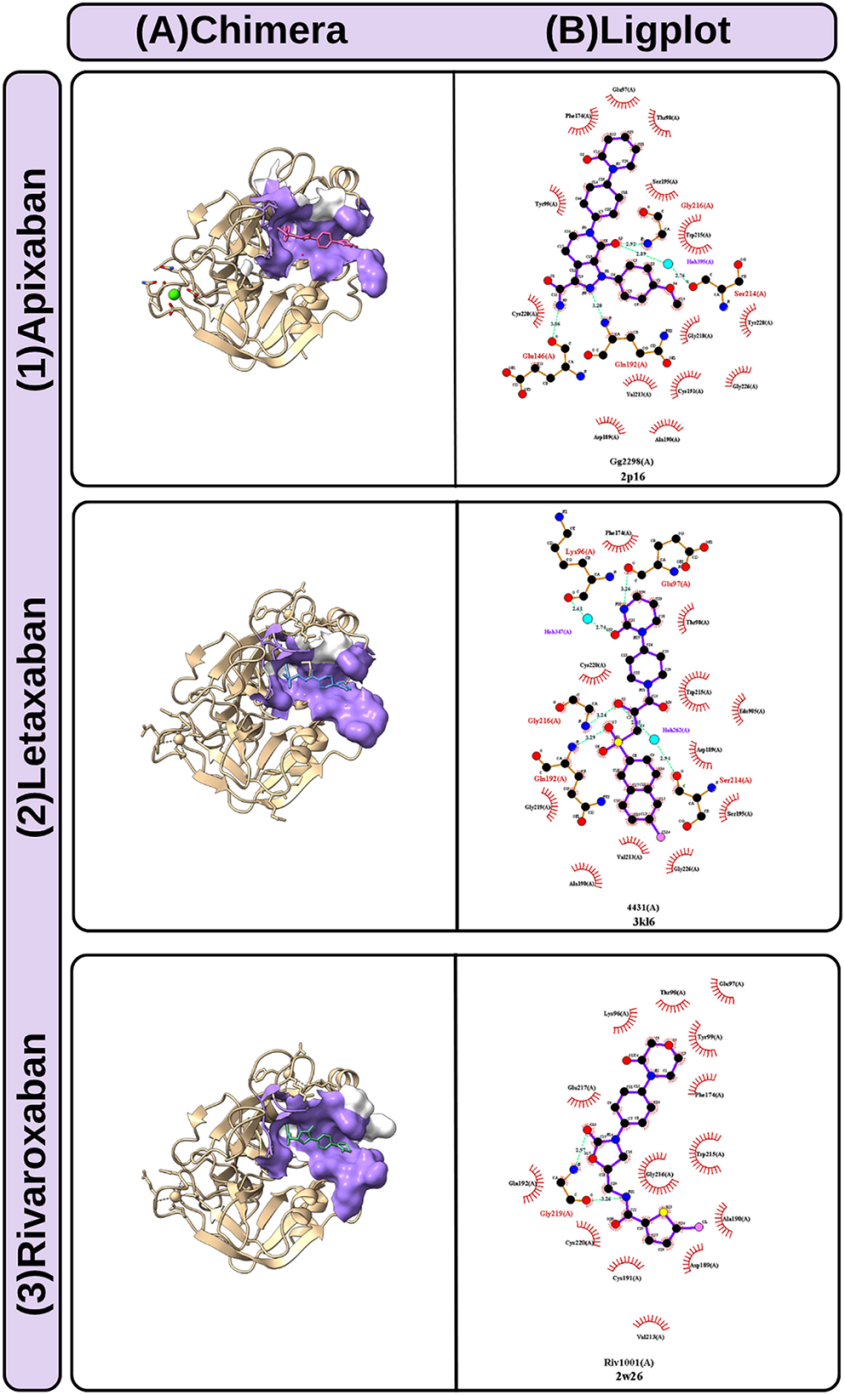
Interaction determination among FXa residues and DOACs. Column A corresponds to molecular visualization in the Chimera interface: FXa interaction interfaces are depicted with the skin surface, and the remaining residues are represented in backbone format; the DOACs molecules are in atomic format. Column B depicts LigPlot results for each DOAC, showing the specific residues and atom interactions.

During the coagulation process, the activation of FX occurs by the cleavage and loss of the activation peptide, reducing its molecular weight from about 68 to about 55 kDa and undergoing a significant conformational change. The performed modeling approach allowed the understanding of domains and chains’ structural distribution and also the verification of modifications caused by the activation process.

### 3.4 Comprehension of DOAC Binding Interaction Sites in FXa and Variants’ Effect on Interaction

Seven nonpathogenic variants of the F10 gene were identified that coincide with the region of interaction of FXa with DOACs according to the search in the EAHAD database and comparison to the analysis of structural sites information on the Chimera interface. Although they present subtle structural variations and since these variants occur in the same binding sites of DOACs, they may interfere with drug–protein interaction. The analyses of the mutation impact predictors (Supporting Information Table S2) indicated that, although classified as nonpathogenic in EAHAD, most of the variants presented a potential deleterious effect. The substitutions altered important characteristics, such as side chain size, electrical charge, and hydrophobicity, with an emphasis on the exchange of negative residues for neutral ones and increased hydrophobicity in six of the seven variants.

Docking simulations using AutoDock Vina provided predicted binding free energies (Δ*G*, kcal/mol) as docking scores for each protein–ligand complex. RMSD values of zero across all tested conditions indicate convergence toward consistent binding modes. Lower Δ*G* values correspond to a more stable complex formation. It should be noted that statistical confidence could not be assessed due to the absence of replicate docking simulations; therefore, the reported Δ*G* values should be interpreted as indicative trends rather than precise energetic differences.

The results indicate that with apixaban and letaxaban, the WT formed more stable complexes than all tested variants (Table 1). However, the scores obtained by the mutated proteins were close to the WT. In the case of apixaban, the p.Tyr319Cys variant was the one with the least stable complex, with a docking score of 23.58% higher than that of the WT. As for letaxaban, the polymorphism p.Glu369Gly presented a less stable complex with a docking score of 22.12% higher than that of the WT. In contrast, in the case of rivaroxaban, the mutated proteins presented a more stable complex than that of the WT, except p.Tyr319Cys, which presented a score of 15.79% higher than that of the WT. It was observed that the largest differences in docking scores were mainly associated with substitutions involving cysteine. In these cases, tyrosine and phenylalanine, both amino acids with cyclic side chains, were replaced by amino acids with linear side chains containing sulfur. Another interesting aspect is that even the variants occurring in the same region of DOAC interactions do not demonstrate interference in docking parameters, which suggests a real in vitro/vivo effect. This means that the studied variants described as nonpathogenic, even if predicted as deleterious in silico, do not impact the interaction with the DOACs. Such findings are important for the development of a point-of-care test, as they suggest that individuals with missense mutations in FX could also benefit from this technology. These results corroborate to expanding the point-of-care applications by minimizing the nondetection profile status.

**Table 1:** Docking Score (Δ*G*, kcal/mol) for FXa Wild Type and Variants with DOACs

	WT^a^	p.Tyr319Asp	p.Tyr319Cys	p.Ala444Thr	p.Phe396Cys	p.Ser419Asn	p.Asp418Gly	p.Glu369Gly
apixaban	–10.6	–9.7	–8,1	–9.1	–9.3	–8,4	–9.3	–8.3
letaxaban	–10.4	–9.2	–9.2	–9.3	–9.2	–8.9	–9.1	–8.4
rivaroxaban	–7.6	–8.6	–6.4	–8.5	–8.8	–8.4	–8.3	–8.8

a WT: wild type. The columns represent the protein structures analyzed, including WT and variants occurring in DOAC binding sites (p.Tyr319Asp, p.Tyr319Cys, p.Ala444Thr, p.Phe396Cys, p.Ser419Asn, p.Asp418Gly, p.Glu369Gly). Each row corresponds to docking scores (Δ*G*, kcal/mol) for different DOACs: apixaban, letaxaban, and rivaroxaban.

## 4 Conclusions and Perspectives

The study demonstrated a promising methodology for modifying SPE by gold electrodeposition and antibody immobilization, enabling the distinction between FX-positive and FX-negative samples in a biological blood matrix. It also showed the capability to discriminate between activated FX in blood samples with preliminary cutoff values established for both negative and positive FX determinations. These thresholds can be further refined for applications in the diagnosis and monitoring of FXa inhibition by DOACs. The immobilization protocol developed in this project, along with the optimized electrochemical measurement range, yielded promising results for the tested samples. Altogether, these findings provide a foundation for the development of a portable device capable of monitoring the FXa activity and DOAC efficacy in clinical settings. This approach may also be adapted for the development of other immunosensors, contributing to advancements in the field. Also, through structural bioinformatics analysis, it was possible to verify that reported amino acid changes in FX-DOAC ligation sites did not disrupt the binding with these drugs.

This work represents an important advancement in the development of an immunosensor for monitoring patients using DOACs. Currently, such monitoring is primarily performed in centralized laboratories through complex analytical methods. The proposed approach aims to provide an accessible point-of-care technological alternative that enables self-monitoring. This strategy has the potential to reduce adverse events associated with both anticoagulant administration and the underlying conditions that necessitate their use.

## Supplementary Material



## Notes

Differential pulse voltammetry (DPV) characterization comparing the basal sensor, the addition of the capture antibody, the antigen, and the complete antigen–antibody complex formation (Figure S1); ten cyclic voltammetry (CV) cycles of the basal graphene–carbon sensor, highlighting the stability achieved at the fifth cycle (Figure S2); raw cyclic voltammetry data for the surface modifications on the screen-printed electrode, including gold electrodeposition, EDC–NHS functionalization, and capture antibody immobilization (Table S1); results of the variant effect prediction algorithms (Table S2) (PDF)

## Abbreviations

AF: atrial fibrillation
Ag/AgCl: silver/silver chloride
AP: activation peptide
AuNSs: gold nanostructures
BSA: bovine serum albumin
cAb: capture antibody
CE: counter electrode
CV: cyclic voltammetry
Δ*E*_p_: difference between peaks
DOACs: direct oral anticoagulants
DPV: differential pulse voltammetry
EAHAD: european association for hemophilia and allied disorders
EDC-NHS: 1-ethyl-3-(3-(dimethylamino)­propyl)-*N*-hydroxysuccinimide
ELISA: enzyme-linked immunosorbent assay
*E*_pa_: anodic peak potential
*E*_pc_: cathodic peak potential
FESEM: field-emission scanning electron microscopy
FX: factor X
FXa: factor X activated
HRP: horseradish peroxidase
ID: identity
*I*_pa_: anodic peak current
*I*_pc_: cathodic peak current
LC-MS/MS: liquid chromatography coupled with mass spectrometry
LOD: limit of detection
MES: 2-(*N*-morpholino)­ethanesulfonic acid
OD: optical density
PBS: phosphate-buffered saline
PDB: Protein Data Bank
POC: point-of-care
PT: prothrombin time
RMSD: root mean square deviation
SPE: screen-printed electrode
SPE_AuNSs: sensor after gold electrodeposition that generates gold nanostructures
SPE_AuNSs_EDCNHS_cAb: sensor after functionalizing with EDC-NHS and immobilization of capture antibody
SPE_nonmodified: unmodified sensor
SP: serine protease domain
TASSER: iterative threading ASSEmbly refinement
TBM: tetramethylbenzidine
TBS-T: Tris-buffered saline with Tween-20
TT: thrombin time
TTBS-BSA: Tris-buffered saline with Tween-20-bovine serum albumin
VKA: vitamin K antagonists
VTE: venous thromboembolism
WE: working electrode
WT: wild type
aPTT: activated partial thromboplastin time
μA·V: peak area
