# Supplementary material for: Development of an Electrochemical Immunosensor for Detecting Coagulation Factor Xa and Perspectives in Monitoring Direct Oral Anticoagulant Therapy
Source: ACS Omega. 2025 Nov 7;10(45):54781–92. doi: 10.1021/acsomega.5c08281 (PMC12631654; doi:10.1021/acsomega.5c08281)
Supplement: Supplementary file 1 [file ao5c08281_si_001.pdf]

Supporting Information - Development of an electrochemical immunosensor for detecting Coagulation Factor Xa and perspectives in monitoring Direct Oral Anticoagulants therapy

Mariana Rost Meireles[1,2,3,] , Julia Konzen Moreira[2,3], Giovana Dalpiaz[1,2], Muriel Schiling Krohn[2], Gabriela Victória de Mello Jantzch[2], Willyan Hasenkamp Carreira[2]*

1. Universidade do Vale do Rio dos Sinos, UNISINOS; Av. Unisinos, 950. São Leopoldo, RS, 93022-000, Brazil.
2. Biosens Development and Biosensors Industry LTDA (BIOSENS); Av. Theodomiro Porto da Fonseca, 3101, São Leopoldo, RS, 93022-715, Brasil.
3. Universidade Federal do Rio Grande do Sul (UFRGS), Chemistry Post Graduation Program; Av. Bento Gonçalves, 9500, Porto Alegre, RS, 91501-970, Brazil.

*Correspondent author: Mariana Rost Meireles,

mrostmeireles@gmail.com / mariana.meireles@biosens.tech/ marianameireles@unisinos.br

KEYWORDS. Direct Oral Anticoagulant (DOAC), Coagulation Factor X, Immunosensor, Point of care, Genetic variants, Electrochemical

Supporting Information Content:

- **Supplementary Figure 1.** Sensors characterization Differential Pulse Voltametry (DPV)
- **Supplementary Figure 2.** Cyclic voltammetry (CV) cycles of a basal graphene-carbon sensor
- **Supplementary Table 1.** Cyclic Voltammetry raw data for the modifications on the SPE surface
- **Supplementary Table 2.** Results of variant effect prediction algorithms

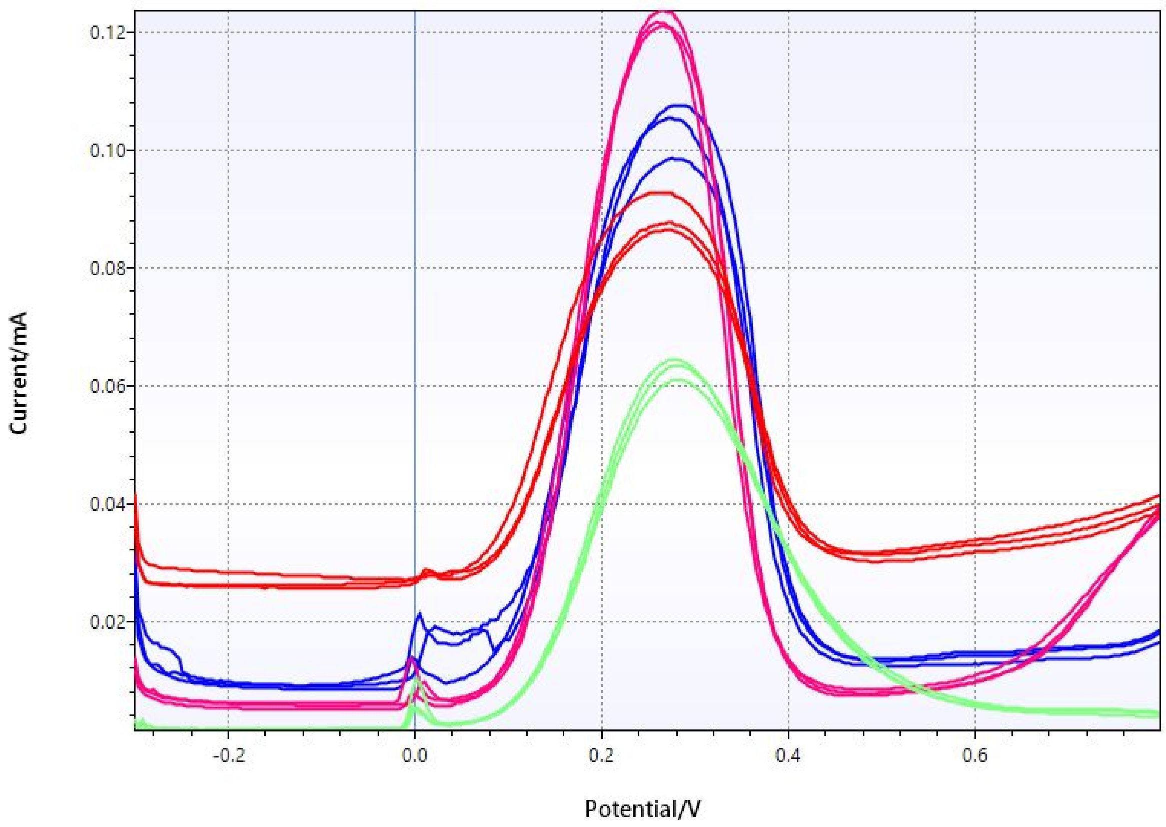

Figure S1 – Supplementary Figure 1. The figure shows the comparison between the

Differential Pulse Voltammetry (DPV) characterization of the basal sensors (green), with the addition of only the capture antibody (blue), only the antigen (pink), and the final step of antigen–antibody complex formation with FXa (red). This step was performed to ensure that the observed result in the complex was not due to the individual components (capture antibody and antigen).

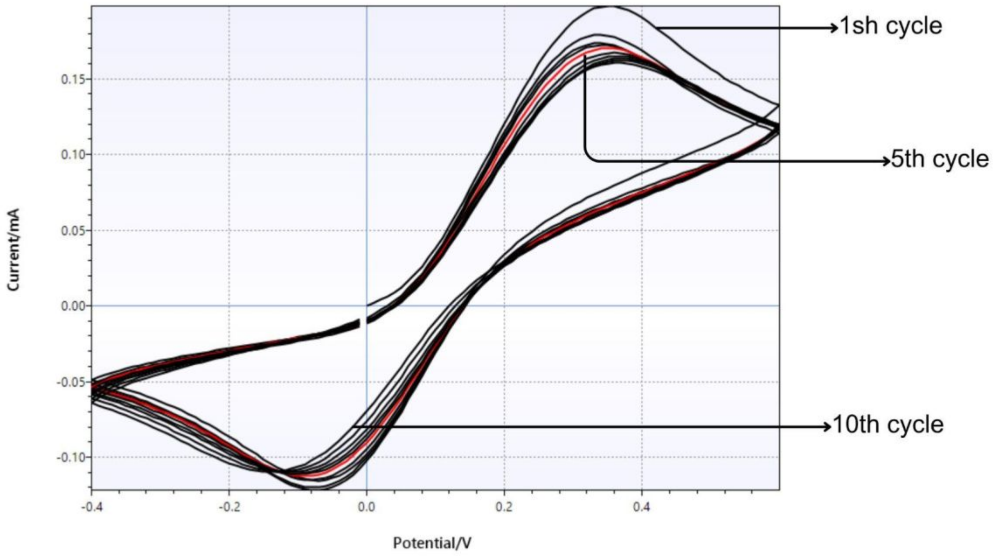

Figure S2 - Supplementary Figure 2. The Figure shows the 10 cyclic voltammetry (CV) cycles of a basal graphene-carbon sensor. The fifth cycle is represented in red, while the remaining nine cycles are shown in black. It can be observed that the cycle with the greatest differentiation pattern is the first one, as highlighted in the image. The mean potential difference between the anodic and cathodic peaks was 153.977 V, with a standard deviation of 15.90. The fifth cycle presented a value very close to the mean (155.051 V), whereas the first and the last cycles were the most divergent, with values of 186.514 V and 133.148 V, respectively. Therefore, the choice of the fifth cycle for the analyses is justified, as it represents the point of greatest stability in the electrochemical measurement.

Table S1. Cyclic Voltammetry raw data for the modifications on the SPE surface—including gold electrodeposition (AuNSs), EDC-NHS functionalization, and capture antibody (cAb) immobilization.

SPE_non modified						SPE_AuNSs						SPE_AuNSs_EDCNHS_cAb					
1		2		2		1		2		3							
V	µA	V	µA	V	µA	V	µA	V	µA	V	µA	V	µA	V	µA	V	µA
0.00	-13.55	0.00	-13.73	0.00	-12.52	0.00	-17.15	0.00	-20.83	0.00	-20.17	0.00	-12.07	0.00	-9.09	0.00	-14.06
0.01	-13.12	0.01	-13.38	0.01	-12.17	0.01	-16.82	0.01	-20.53	0.01	-19.87	0.01	-11.71	0.01	-8.77	0.01	-13.72
0.02	-12.66	0.02	-13.01	0.02	-11.80	0.02	-16.48	0.02	-20.21	0.02	-19.56	0.02	-11.28	0.02	-8.48	0.02	-13.39
0.03	-9.80	0.03	-11.44	0.03	-10.03	0.03	-14.21	0.03	-18.51	0.03	-17.94	0.03	-10.39	0.03	-7.61	0.03	-12.83
0.04	-10.17	0.04	-11.85	0.04	-10.27	0.04	-10.51	0.04	-16.46	0.04	-15.71	0.04	-10.20	0.04	-7.08	0.04	-12.47
0.05	-10.15	0.05	-11.49	0.05	-10.01	0.05	-12.83	0.05	-17.91	0.05	-16.74	0.05	-9.71	0.05	-6.92	0.05	-11.76
0.06	-10.41	0.06	-11.43	0.06	-9.94	0.06	-13.83	0.06	-18.16	0.06	-17.27	0.06	-9.42	0.06	-6.78	0.06	-10.74
0.07	-10.24	0.07	-11.08	0.07	-9.80	0.07	-14.14	0.07	-18.15	0.07	-17.36	0.07	-9.28	0.07	-6.67	0.07	-10.97
0.08	-9.83	0.08	-10.61	0.08	-9.39	0.08	-13.99	0.08	-17.89	0.08	-17.25	0.08	-8.92	0.08	-6.35	0.08	-11.13
0.09	-9.32	0.09	-10.09	0.09	-8.93	0.09	-13.45	0.09	-17.38	0.09	-16.74	0.09	-8.35	0.09	-5.86	0.09	-10.75
0.10	-8.66	0.10	-9.49	0.10	-8.41	0.10	-12.70	0.10	-16.65	0.10	-16.06	0.10	-7.65	0.10	-5.28	0.10	-10.20
0.11	-8.08	0.11	-8.78	0.11	-7.77	0.11	-11.75	0.11	-15.73	0.11	-15.16	0.11	-6.74	0.11	-4.53	0.11	-9.46
0.12	-7.31	0.12	-7.96	0.12	-7.03	0.12	-10.56	0.12	-14.54	0.12	-14.00	0.12	-5.60	0.12	-3.51	0.12	-8.52
0.13	-6.40	0.13	-6.95	0.13	-6.11	0.13	-8.97	0.13	-12.94	0.13	-12.43	0.13	-4.00	0.13	-2.05	0.13	-7.16
0.14	-5.29	0.14	-5.71	0.14	-5.01	0.14	-6.88	0.14	-10.84	0.14	-10.37	0.14	-1.87	0.14	-0.12	0.14	-5.34
0.15	-3.93	0.15	-4.15	0.15	-3.64	0.15	-4.13	0.15	-8.03	0.15	-7.57	0.15	1.07	0.15	2.54	0.15	-2.80
0.16	-2.28	0.16	-2.26	0.16	-1.94	0.16	-0.55	0.16	-4.33	0.16	-3.89	0.16	4.89	0.16	6.02	0.16	0.53
0.17	-0.24	0.17	0.13	0.17	0.18	0.17	4.14	0.17	0.53	0.17	0.96	0.17	9.94	0.17	10.60	0.17	5.08
0.18	2.20	0.18	3.00	0.18	2.77	0.18	9.94	0.18	6.57	0.18	7.03	0.18	16.12	0.18	16.21	0.18	10.78
0.19	5.24	0.19	6.55	0.19	6.01	0.19	17.18	0.19	14.14	0.19	14.68	0.19	23.84	0.19	23.26	0.19	18.06
0.20	8.78	0.20	10.70	0.20	9.83	0.20	25.51	0.20	22.89	0.20	23.53	0.20	32.64	0.20	31.29	0.20	26.50
0.21	12.98	0.21	15.61	0.21	14.40	0.21	35.17	0.21	33.05	0.21	33.86	0.21	42.78	0.21	40.62	0.21	36.44
0.22	17.85	0.22	21.28	0.22	19.77	0.22	46.06	0.22	44.52	0.22	45.52	0.22	54.21	0.22	51.19	0.22	47.76
0.23	23.32	0.23	27.61	0.23	25.83	0.23	57.81	0.23	56.90	0.23	58.13	0.23	66.54	0.23	62.59	0.23	60.10
0.24	29.40	0.24	34.62	0.24	32.61	0.24	70.46	0.24	70.23	0.24	71.76	0.24	79.73	0.24	74.80	0.24	73.52
0.25	36.03	0.25	42.26	0.25	40.06	0.25	83.76	0.25	84.24	0.25	86.09	0.25	93.69	0.25	87.78	0.25	87.73
0.26	43.12	0.26	50.43	0.26	48.08	0.26	97.61	0.26	98.80	0.26	100.99	0.26	108.26	0.26	101.32	0.26	102.63
0.27	50.46	0.27	58.93	0.27	56.43	0.27	111.58	0.27	113.45	0.27	115.92	0.27	122.93	0.27	115.04	0.27	117.68
0.28	58.12	0.28	67.76	0.28	65.20	0.28	125.81	0.28	128.33	0.28	131.15	0.28	138.00	0.28	129.11	0.28	133.17
0.29	65.96	0.29	76.85	0.29	74.16	0.29	140.18	0.29	143.24	0.29	146.37	0.29	153.22	0.29	143.40	0.29	148.86

0.30	73.82	0.30	86.00	0.30	83.20	0.30	154.32	0.30	157.80	0.30	161.26	0.30	168.38	0.30	157.57	0.30	164.39
0.31	81.68	0.31	95.17	0.31	92.24	0.31	168.16	0.31	171.96	0.31	175.71	0.31	183.36	0.31	171.63	0.31	179.73
0.32	89.37	0.32	104.16	0.32	101.05	0.32	181.25	0.32	185.23	0.32	189.18	0.32	197.73	0.32	185.18	0.32	194.36
0.33	96.97	0.33	113.05	0.33	109.74	0.33	193.70	0.33	197.61	0.33	201.73	0.33	211.55	0.33	198.35	0.33	208.34
0.34	104.23	0.34	121.50	0.34	117.96	0.34	204.57	0.34	208.11	0.34	212.13	0.34	224.03	0.34	210.36	0.34	220.88
0.35	111.02	0.35	129.33	0.35	125.60	0.35	213.39	0.35	216.10	0.35	220.20	0.35	234.65	0.35	220.87	0.35	231.29
0.36	117.64	0.36	136.86	0.36	132.92	0.36	220.40	0.36	221.57	0.36	225.36	0.36	243.73	0.36	230.15	0.36	240.14
0.37	123.74	0.37	143.63	0.37	139.53	0.37	224.15	0.37	223.76	0.37	226.62	0.37	249.95	0.37	236.78	0.37	245.58
0.38	129.35	0.38	149.62	0.38	145.38	0.38	224.24	0.38	221.49	0.38	223.83	0.38	252.46	0.38	240.59	0.38	247.03
0.39	134.40	0.39	154.90	0.39	150.59	0.39	220.02	0.39	214.97	0.39	216.76	0.39	250.15	0.39	240.42	0.39	243.56
0.40	138.66	0.40	159.33	0.40	155.01	0.40	211.98	0.40	205.32	0.40	206.80	0.40	242.57	0.40	235.73	0.40	235.10
0.41	142.96	0.41	162.84	0.41	158.57	0.41	201.40	0.41	194.24	0.41	195.61	0.41	229.97	0.41	226.37	0.41	222.64
0.42	146.45	0.42	165.41	0.42	161.44	0.42	190.23	0.42	183.41	0.42	184.81	0.42	215.03	0.42	213.85	0.42	208.89
0.43	149.01	0.43	166.93	0.43	163.32	0.43	179.71	0.43	173.54	0.43	174.98	0.43	200.72	0.43	200.66	0.43	196.08
0.44	151.47	0.44	167.66	0.44	164.52	0.44	170.51	0.44	164.96	0.44	166.40	0.44	188.73	0.44	188.93	0.44	185.22
0.45	153.23	0.45	167.52	0.45	164.93	0.45	162.48	0.45	157.45	0.45	158.90	0.45	178.80	0.45	178.99	0.45	176.03
0.46	154.38	0.46	166.52	0.46	164.59	0.46	155.49	0.46	150.87	0.46	152.31	0.46	170.48	0.46	170.57	0.46	168.18
0.47	155.03	0.47	164.92	0.47	163.71	0.47	149.37	0.47	145.09	0.47	146.51	0.47	163.47	0.47	163.48	0.47	161.48
0.48	155.18	0.48	162.72	0.48	162.28	0.48	143.97	0.48	139.94	0.48	141.35	0.48	157.41	0.48	157.34	0.48	155.63
0.49	154.81	0.49	160.04	0.49	160.36	0.49	139.15	0.49	135.33	0.49	136.73	0.49	152.13	0.49	151.99	0.49	150.48
0.50	153.97	0.50	156.99	0.50	158.05	0.50	134.82	0.50	131.17	0.50	132.55	0.50	147.44	0.50	147.26	0.50	145.91
0.51	152.77	0.51	153.72	0.51	155.47	0.51	130.91	0.51	127.38	0.51	128.76	0.51	143.30	0.51	143.09	0.51	141.81
0.52	151.29	0.52	150.36	0.52	152.73	0.52	127.37	0.52	123.95	0.52	125.33	0.52	139.61	0.52	139.39	0.52	138.16
0.53	149.51	0.53	146.91	0.53	149.84	0.53	124.13	0.53	120.80	0.53	122.17	0.53	136.27	0.53	136.04	0.53	134.83
0.54	147.54	0.54	143.50	0.54	146.89	0.54	121.16	0.54	117.91	0.54	119.28	0.54	133.26	0.54	133.05	0.54	131.81
0.55	145.37	0.55	140.11	0.55	143.91	0.55	118.43	0.55	115.23	0.55	116.61	0.55	130.48	0.55	130.28	0.55	129.04
0.56	143.10	0.56	136.84	0.56	140.98	0.56	115.92	0.56	112.75	0.56	114.15	0.56	127.99	0.56	127.81	0.56	126.52
0.57	140.71	0.57	133.67	0.57	138.04	0.57	113.59	0.57	110.46	0.57	111.85	0.57	125.66	0.57	125.52	0.57	124.16
0.58	138.30	0.58	130.63	0.58	135.21	0.58	111.43	0.58	108.33	0.58	109.73	0.58	123.56	0.58	123.45	0.58	122.01
0.59	135.89	0.59	127.75	0.59	132.48	0.59	109.43	0.59	106.35	0.59	107.76	0.59	121.63	0.59	121.57	0.59	120.03
0.60	133.49	0.60	125.00	0.60	129.83	0.60	107.56	0.60	104.50	0.60	105.92	0.60	119.86	0.60	119.85	0.60	118.20
0.61	131.15	0.61	122.40	0.61	127.30	0.61	105.84	0.61	102.78	0.61	104.22	0.61	118.24	0.61	118.31	0.61	116.55
0.62	128.84	0.62	119.92	0.62	124.88	0.62	104.21	0.62	101.17	0.62	102.61	0.62	116.73	0.62	116.88	0.62	115.01
0.63	126.63	0.63	117.59	0.63	122.55	0.63	102.68	0.63	99.66	0.63	101.11	0.63	115.33	0.63	115.55	0.63	113.60
0.64	124.47	0.64	115.37	0.64	120.34	0.64	101.27	0.64	98.26	0.64	99.71	0.64	114.02	0.64	114.36	0.64	112.30
0.65	122.45	0.65	113.28	0.65	118.23	0.65	99.94	0.65	96.93	0.65	98.40	0.65	112.80	0.65	113.25	0.65	111.07
0.66	120.48	0.66	111.28	0.66	116.21	0.66	98.67	0.66	95.69	0.66	97.17	0.66	111.64	0.66	112.17	0.66	109.89
0.67	118.61	0.67	109.39	0.65	111.33	0.67	97.51	0.67	94.54	0.67	96.02	0.67	110.63	0.67	111.23	0.67	108.82
0.68	116.87	0.66	105.33	0.64	107.51	0.68	96.43	0.68	93.48	0.68	94.96	0.68	109.77	0.68	110.43	0.68	107.88
0.67	110.46	0.65	102.13	0.63	104.22	0.69	95.44	0.69	92.49	0.69	93.98	0.69	109.02	0.69	109.71	0.69	107.04
0.66	105.86	0.64	99.35	0.62	101.25	0.70	94.53	0.70	91.56	0.70	93.07	0.70	108.42	0.70	109.15	0.70	106.34
0.65	102.04	0.63	96.83	0.61	98.54	0.71	93.69	0.71	90.71	0.71	92.21	0.71	107.93	0.71	108.67	0.71	105.75

0.64	98.67	0.62	94.51	0.60	95.98	0.72	92.95	0.72	89.94	0.72	91.46	0.72	107.64	0.72	108.42	0.72	105.34
0.63	95.64	0.61	92.33	0.59	93.58	0.73	92.27	0.73	89.21	0.73	90.74	0.73	107.42	0.73	108.22	0.73	105.01
0.62	92.86	0.60	90.26	0.58	91.28	0.74	91.67	0.74	88.55	0.74	90.08	0.74	107.36	0.74	108.18	0.74	104.86
0.61	90.28	0.59	88.29	0.57	89.08	0.75	91.17	0.75	87.98	0.75	89.52	0.75	107.56	0.75	108.40	0.75	104.95
0.60	87.84	0.58	86.40	0.56	86.94	0.76	90.73	0.76	87.46	0.76	89.02	0.76	107.91	0.74	97.24	0.76	105.18
0.59	85.51	0.57	84.57	0.55	84.85	0.77	90.38	0.75	83.86	0.75	85.53	0.77	108.46	0.73	90.02	0.75	96.52
0.58	83.28	0.56	82.78	0.54	82.81	0.78	90.10	0.74	81.41	0.74	83.12	0.76	97.91	0.72	84.56	0.74	90.74
0.57	81.14	0.55	81.00	0.53	80.78	0.77	85.87	0.73	79.48	0.73	81.21	0.75	90.90	0.71	80.12	0.73	86.41
0.56	79.06	0.54	79.27	0.52	78.79	0.76	83.01	0.72	77.85	0.72	79.59	0.74	85.55	0.70	76.51	0.72	82.89
0.55	77.00	0.53	77.54	0.51	76.79	0.75	80.79	0.71	76.40	0.71	78.15	0.73	81.34	0.69	73.41	0.71	79.90
0.54	75.01	0.52	75.82	0.50	74.81	0.74	78.94	0.70	75.13	0.70	76.89	0.72	77.83	0.68	70.77	0.70	77.39
0.53	73.01	0.51	74.10	0.49	72.83	0.73	77.37	0.69	73.97	0.69	75.73	0.71	74.80	0.67	68.41	0.69	75.16
0.52	71.05	0.50	72.38	0.48	70.80	0.72	75.99	0.68	72.91	0.68	74.67	0.70	72.25	0.66	66.38	0.68	73.21
0.51	69.10	0.49	70.63	0.47	68.74	0.71	74.74	0.67	71.93	0.67	73.68	0.69	69.99	0.65	64.56	0.67	71.43
0.50	67.17	0.48	68.84	0.46	66.66	0.70	73.62	0.66	71.01	0.66	72.77	0.68	67.99	0.64	62.88	0.66	69.84
0.49	65.23	0.47	67.02	0.45	64.54	0.69	72.58	0.65	70.16	0.65	71.91	0.67	66.18	0.63	61.36	0.65	68.40
0.48	63.26	0.46	65.13	0.44	62.33	0.68	71.64	0.64	69.34	0.64	71.09	0.66	64.59	0.62	59.99	0.64	67.04
0.47	61.25	0.45	63.20	0.43	60.06	0.67	70.75	0.63	68.58	0.63	70.32	0.65	63.14	0.61	58.73	0.63	65.79
0.46	59.24	0.44	61.18	0.42	57.72	0.66	69.93	0.62	67.85	0.62	69.58	0.64	61.77	0.60	57.56	0.62	64.63
0.45	57.20	0.43	59.07	0.41	55.22	0.65	69.16	0.61	67.16	0.61	68.87	0.63	60.52	0.59	56.49	0.61	63.54
0.44	55.10	0.42	56.88	0.40	52.60	0.64	68.41	0.60	66.48	0.60	68.20	0.62	59.39	0.58	55.48	0.60	62.51
0.43	52.94	0.41	54.51	0.39	49.82	0.63	67.72	0.59	65.83	0.59	67.54	0.61	58.34	0.57	54.56	0.59	61.55
0.42	50.76	0.40	51.99	0.38	46.87	0.62	67.05	0.58	65.21	0.58	66.90	0.60	57.35	0.56	53.70	0.58	60.65
0.41	48.43	0.39	49.27	0.37	43.65	0.61	66.41	0.57	64.59	0.57	66.28	0.59	56.44	0.55	52.85	0.57	59.78
0.40	46.02	0.38	46.36	0.36	40.15	0.60	65.79	0.56	64.01	0.56	65.68	0.58	55.58	0.54	52.08	0.56	58.99
0.39	43.48	0.37	43.14	0.35	36.28	0.59	65.20	0.55	63.42	0.55	65.09	0.57	54.78	0.53	51.33	0.55	58.20
0.38	40.80	0.36	39.62	0.34	32.13	0.58	64.62	0.54	62.85	0.54	64.51	0.56	54.03	0.52	50.63	0.54	57.47
0.37	37.90	0.35	35.68	0.33	27.48	0.57	64.06	0.53	62.30	0.53	63.93	0.55	53.29	0.51	49.95	0.53	56.74
0.36	34.79	0.34	31.43	0.32	22.29	0.56	63.51	0.52	61.74	0.52	63.36	0.54	52.61	0.50	49.33	0.52	56.07
0.35	31.35	0.33	26.64	0.31	16.66	0.55	62.97	0.51	61.20	0.51	62.79	0.53	51.95	0.49	48.71	0.51	55.43
0.34	27.70	0.32	21.29	0.30	10.45	0.54	62.44	0.50	60.66	0.50	62.23	0.52	51.34	0.48	48.09	0.50	54.83
0.33	23.60	0.31	15.49	0.29	3.76	0.53	61.91	0.49	60.11	0.49	61.67	0.51	50.73	0.47	47.46	0.49	54.22
0.32	19.03	0.30	9.12	0.28	-3.45	0.52	61.39	0.48	59.55	0.48	61.09	0.50	50.18	0.46	46.82	0.48	53.62
0.31	14.08	0.29	2.25	0.27	-11.04	0.51	60.87	0.47	58.96	0.47	60.48	0.49	49.61	0.45	46.20	0.47	53.00
0.30	8.64	0.28	-5.12	0.26	-18.94	0.50	60.34	0.46	58.36	0.46	59.86	0.48	49.04	0.44	45.48	0.46	52.38
0.29	2.74	0.27	-12.89	0.25	-27.22	0.49	59.81	0.45	57.71	0.45	59.19	0.47	48.47	0.43	44.70	0.45	51.74
0.28	-3.60	0.26	-20.97	0.24	-35.62	0.48	59.25	0.44	57.01	0.44	58.46	0.46	47.89	0.42	43.84	0.44	51.02
0.27	-10.32	0.25	-29.43	0.23	-44.01	0.47	58.69	0.43	56.21	0.43	57.64	0.45	47.30	0.41	42.73	0.43	50.21
0.26	-17.30	0.24	-38.04	0.22	-52.27	0.46	58.08	0.42	55.32	0.42	56.71	0.44	46.65	0.40	41.41	0.42	49.32
0.25	-24.65	0.23	-46.69	0.21	-60.39	0.45	57.43	0.41	54.22	0.41	55.60	0.43	45.89	0.39	39.72	0.41	48.15
0.24	-32.12	0.22	-55.26	0.20	-68.03	0.44	56.71	0.40	52.89	0.40	54.24	0.42	45.06	0.38	37.60	0.40	46.71
0.23	-39.61	0.21	-63.79	0.19	-75.12	0.43	55.90	0.39	51.21	0.39	52.55	0.41	43.98	0.37	34.79	0.39	44.87

0.22	-47.01	0.20	-71.88	0.18	-81.78	0.42	54.97	0.38	49.11	0.38	50.43	0.40	42.67	0.36	31.17	0.38	42.50
0.21	-54.31	0.19	-79.49	0.17	-87.58	0.41	53.83	0.37	46.39	0.37	47.70	0.39	40.98	0.35	26.50	0.37	39.34
0.20	-61.30	0.18	-86.71	0.16	-92.71	0.40	52.45	0.36	42.88	0.36	44.20	0.38	38.82	0.34	20.95	0.36	35.25
0.19	-67.80	0.17	-93.10	0.15	-96.92	0.39	50.72	0.35	38.37	0.35	39.68	0.37	35.95	0.33	14.06	0.35	29.92
0.18	-74.00	0.16	-98.81	0.14	-100.28	0.38	48.57	0.34	32.90	0.34	34.23	0.36	32.22	0.32	5.84	0.34	23.55
0.17	-79.52	0.15	-103.46	0.13	-102.59	0.37	45.78	0.33	26.08	0.33	27.42	0.35	27.40	0.31	-3.39	0.33	15.66
0.16	-84.55	0.14	-107.51	0.12	-104.51	0.36	42.25	0.32	17.84	0.32	19.20	0.34	21.61	0.30	-13.80	0.32	6.24
0.15	-88.84	0.13	-110.37	0.11	-105.34	0.35	37.75	0.31	8.50	0.31	9.85	0.33	14.37	0.29	-25.17	0.31	-4.28
0.14	-92.50	0.12	-112.24	0.10	-105.46	0.34	32.38	0.30	-2.14	0.30	-0.84	0.32	5.73	0.28	-37.44	0.30	-16.13
0.13	-95.41	0.11	-113.06	0.09	-104.92	0.33	25.73	0.29	-13.82	0.29	-12.60	0.31	-3.98	0.27	-50.33	0.29	-29.00
0.12	-96.92	0.10	-113.03	0.08	-103.80	0.32	17.79	0.28	-26.51	0.28	-25.39	0.30	-15.03	0.26	-63.81	0.28	-42.85
0.11	-99.47	0.09	-112.18	0.07	-102.20	0.31	8.83	0.27	-39.90	0.27	-38.92	0.29	-27.06	0.25	-77.87	0.27	-57.36
0.10	-100.59	0.08	-110.62	0.06	-100.16	0.30	-1.33	0.26	-53.79	0.26	-52.99	0.28	-40.10	0.24	-92.19	0.26	-72.41
0.09	-101.08	0.07	-108.49	0.05	-97.86	0.29	-12.44	0.25	-68.39	0.25	-67.75	0.27	-53.82	0.23	-106.64	0.25	-88.05
0.08	-101.09	0.06	-105.86	0.04	-95.35	0.28	-24.46	0.24	-83.18	0.24	-82.71	0.26	-68.14	0.22	-121.01	0.24	-103.87
0.07	-100.63	0.05	-102.91	0.03	-92.65	0.27	-37.12	0.23	-97.95	0.23	-97.68	0.25	-83.11	0.21	-135.37	0.23	-119.68
0.06	-99.72	0.04	-99.77	0.02	-89.93	0.26	-50.27	0.22	-112.53	0.22	-112.42	0.24	-98.36	0.20	-149.17	0.22	-135.26
0.05	-98.52	0.03	-96.46	0.01	-87.16	0.25	-64.14	0.21	-126.90	0.21	-126.94	0.23	-113.73	0.19	-162.22	0.21	-150.65
0.04	-97.03	0.02	-93.16	0.00	-84.40	0.24	-78.14	0.20	-140.44	0.20	-140.56	0.22	-129.02	0.18	-174.81	0.20	-165.11
0.03	-95.27	0.01	-89.86	-0.01	-81.65	0.23	-92.19	0.19	-152.86	0.19	-152.99	0.21	-144.30	0.17	-185.86	0.19	-178.42
0.02	-93.38	0.00	-86.64	-0.02	-78.98	0.22	-106.11	0.18	-164.28	0.18	-164.34	0.20	-158.95	0.16	-195.56	0.18	-190.67
0.01	-91.34	-0.01	-83.48	-0.03	-76.38	0.21	-119.87	0.17	-173.57	0.17	-173.46	0.19	-172.79	0.15	-202.96	0.17	-200.56
0.00	-89.20	-0.02	-80.46	-0.04	-73.87	0.20	-132.94	0.16	-180.56	0.16	-180.54	0.18	-186.04	0.14	-207.66	0.16	-207.99
-0.01	-86.95	-0.03	-77.57	-0.05	-71.46	0.19	-145.08	0.15	-184.64	0.15	-184.01	0.17	-197.56	0.13	-208.22	0.15	-212.05
-0.02	-84.70	-0.04	-74.80	-0.06	-69.15	0.18	-156.42	0.14	-184.95	0.14	-183.70	0.16	-207.48	0.12	-204.08	0.14	-211.49
-0.03	-82.42	-0.05	-72.18	-0.07	-66.96	0.17	-165.94	0.13	-180.86	0.13	-179.27	0.15	-214.66	0.11	-194.66	0.13	-205.15
-0.04	-80.15	-0.06	-69.70	-0.08	-64.85	0.16	-173.61	0.12	-173.03	0.12	-171.31	0.14	-218.78	0.10	-181.72	0.12	-193.65
-0.05	-77.91	-0.07	-67.37	-0.09	-62.84	0.15	-178.52	0.11	-162.70	0.11	-161.06	0.13	-218.23	0.09	-167.95	0.11	-179.13
-0.06	-75.72	-0.08	-65.14	-0.10	-60.94	0.14	-180.30	0.10	-151.86	0.10	-150.38	0.12	-212.17	0.08	-155.61	0.10	-165.05

-0.07	-73.59	-0.09	-63.05	-0.11	-59.14	0.13	-178.09	0.09	-141.65	0.09	-140.30	0.11	-200.35	0.07	-145.27	0.09	-152.88
-0.08	-71.50	-0.10	-61.07	-0.12	-57.43	0.12	-172.06	0.08	-132.54	0.08	-131.28	0.10	-185.25	0.06	-136.60	0.08	-142.75
-0.09	-69.49	-0.11	-59.20	-0.13	-55.95	0.11	-162.75	0.07	-124.57	0.07	-123.35	0.09	-170.22	0.05	-129.37	0.07	-134.25
-0.10	-67.56	-0.12	-57.43	-0.14	-55.31	0.10	-152.09	0.06	-117.58	0.06	-116.37	0.08	-157.40	0.04	-123.19	0.06	-127.01
-0.11	-65.70	-0.13	-55.81	-0.15	-55.19	0.09	-141.62	0.05	-111.45	0.05	-110.25	0.07	-146.88	0.03	-117.77	0.05	-120.78
-0.12	-63.90	-0.14	-54.54	-0.16	-55.92	0.08	-132.12	0.04	-106.04	0.04	-104.83	0.06	-138.15	0.02	-113.13	0.04	-115.36
-0.13	-62.20	-0.15	-53.48	-0.17	-54.96	0.07	-123.79	0.03	-101.20	0.03	-99.98	0.05	-130.85	0.01	-108.99	0.03	-110.55
-0.14	-60.76	-0.16	-52.98	-0.18	-51.48	0.06	-116.49	0.02	-96.90	0.02	-95.64	0.04	-124.60	0.00	-105.30	0.02	-106.33
-0.15	-60.11	-0.17	-51.90	-0.19	-48.72	0.05	-110.14	0.01	-92.99	0.01	-91.72	0.03	-119.14	-0.01	-101.96	0.01	-102.50
-0.16	-60.12	-0.18	-49.94	-0.20	-47.21	0.04	-104.54	0.00	-89.45	0.00	-88.17	0.02	-114.40	-0.02	-98.97	0.00	-99.08
-0.17	-59.17	-0.19	-48.09	-0.21	-46.13	0.03	-99.55	-0.01	-86.22	-0.01	-84.91	0.01	-110.19	-0.03	-96.23	-0.01	-95.93
-0.18	-59.23	-0.20	-46.56	-0.22	-44.99	0.02	-95.11	-0.02	-83.27	-0.02	-81.93	0.00	-106.42	-0.04	-93.73	-0.02	-93.09
-0.19	-56.11	-0.21	-45.72	-0.23	-43.41	0.01	-91.12	-0.03	-80.54	-0.03	-79.18	-0.01	-103.02	-0.05	-91.45	-0.03	-90.48
-0.20	-53.49	-0.22	-44.30	-0.24	-42.46	0.00	-87.51	-0.04	-78.02	-0.04	-76.66	-0.02	-99.95	-0.06	-89.35	-0.04	-88.09
-0.21	-51.85	-0.23	-43.14	-0.25	-41.58	-0.01	-84.21	-0.05	-75.73	-0.05	-74.37	-0.03	-97.15	-0.07	-87.47	-0.05	-85.93
-0.22	-51.04	-0.24	-42.29	-0.26	-40.84	-0.02	-81.20	-0.06	-73.67	-0.06	-72.33	-0.04	-94.58	-0.08	-85.69	-0.06	-83.97
-0.23	-49.87	-0.25	-41.67	-0.27	-40.18	-0.03	-78.43	-0.07	-71.83	-0.07	-70.59	-0.05	-92.23	-0.09	-84.03	-0.07	-82.20
-0.24	-48.73	-0.26	-41.09	-0.28	-39.49	-0.04	-75.89	-0.08	-70.13	-0.08	-68.94	-0.06	-90.07	-0.10	-82.46	-0.08	-80.52
-0.25	-47.07	-0.27	-40.27	-0.29	-38.47	-0.05	-73.57	-0.09	-68.43	-0.09	-67.12	-0.07	-88.15	-0.11	-81.02	-0.09	-78.87
-0.26	-46.43	-0.28	-38.85	-0.30	-37.91	-0.06	-71.49	-0.10	-66.68	-0.10	-65.44	-0.08	-86.37	-0.12	-79.65	-0.10	-77.25
-0.27	-45.82	-0.29	-38.48	-0.31	-37.25	-0.07	-69.71	-0.11	-64.98	-0.11	-63.64	-0.09	-84.63	-0.13	-78.43	-0.11	-75.70
-0.28	-45.13	-0.30	-37.71	-0.32	-36.62	-0.08	-68.12	-0.12	-63.48	-0.12	-62.19	-0.10	-82.95	-0.14	-77.34	-0.12	-74.25
-0.29	-44.71	-0.31	-37.17	-0.33	-36.73	-0.09	-66.49	-0.13	-62.25	-0.13	-61.13	-0.11	-81.41	-0.15	-76.33	-0.13	-72.91
-0.30	-44.50	-0.32	-36.66	-0.34	-36.63	-0.10	-64.83	-0.14	-61.26	-0.14	-60.08	-0.12	-79.94	-0.16	-75.43	-0.14	-71.58
-0.31	-43.78	-0.33	-35.82	-0.35	-36.33	-0.11	-63.11	-0.15	-60.38	-0.15	-58.92	-0.13	-78.62	-0.17	-74.59	-0.15	-70.33
-0.32	-42.98	-0.34	-35.93	-0.36	-35.96	-0.12	-61.56	-0.16	-59.29	-0.16	-57.88	-0.14	-77.40	-0.18	-73.82	-0.16	-69.26
-0.33	-42.92	-0.35	-35.25	-0.37	-36.00	-0.13	-60.44	-0.17	-58.56	-0.17	-56.88	-0.15	-76.22	-0.19	-73.14	-0.17	-68.30
-0.34	-43.00	-0.36	-34.71	-0.38	-34.93	-0.14	-59.97	-0.18	-57.07	-0.18	-55.61	-0.16	-75.12	-0.20	-72.44	-0.18	-67.43
-0.35	-43.06	-0.37	-34.14	-0.39	-34.54	-0.15	-59.15	-0.19	-55.66	-0.19	-54.08	-0.17	-74.19	-0.21	-71.63	-0.19	-66.56
-0.36	-43.06	-0.38	-34.91	-0.40	-35.13	-0.16	-58.15	-0.20	-54.40	-0.20	-52.52	-0.18	-73.43	-0.22	-70.78	-0.20	-65.63
-0.37	-43.34	-0.39	-34.39	-0.39	-32.54	-0.17	-57.40	-0.21	-52.70	-0.21	-51.27	-0.19	-72.59	-0.23	-70.26	-0.21	-64.88
-0.38	-43.53	-0.40	-33.78	-0.38	-31.23	-0.18	-56.46	-0.22	-51.62	-0.22	-49.99	-0.20	-71.80	-0.24	-69.54	-0.22	-64.17
-0.39	-43.73	-0.39	-32.12	-0.37	-30.54	-0.19	-55.01	-0.23	-50.68	-0.23	-49.06	-0.21	-70.96	-0.25	-68.98	-0.23	-63.41
-0.40	-41.24	-0.38	-30.84	-0.36	-29.30	-0.20	-53.62	-0.24	-49.84	-0.24	-48.21	-0.22	-70.24	-0.26	-68.45	-0.24	-62.79
-0.39	-40.12	-0.37	-29.89	-0.35	-28.15	-0.21	-51.72	-0.25	-49.11	-0.25	-47.43	-0.23	-73.82	-0.27	-68.13	-0.25	-62.21
-0.38	-37.59	-0.36	-28.91	-0.34	-27.57	-0.22	-50.16	-0.26	-48.44	-0.26	-46.76	-0.24	-71.84	-0.28	-68.05	-0.26	-61.81

-0.37	-35.30	-0.35	-28.01	-0.33	-26.39	-0.23	-48.94	-0.27	-48.06	-0.27	-46.15	-0.25	-69.97	-0.29	-68.06	-0.27	-61.56
-0.36	-33.63	-0.34	-27.22	-0.32	-25.68	-0.24	-48.03	-0.28	-47.28	-0.28	-45.55	-0.26	-68.33	-0.30	-68.17	-0.28	-61.54
-0.35	-32.16	-0.33	-26.48	-0.31	-24.93	-0.25	-47.52	-0.29	-46.80	-0.29	-45.06	-0.27	-68.95	-0.31	-68.41	-0.29	-61.48
-0.34	-30.94	-0.32	-25.79	-0.30	-24.28	-0.26	-46.57	-0.30	-46.42	-0.30	-44.65	-0.28	-67.45	-0.32	-68.88	-0.30	-61.26
-0.33	-29.56	-0.31	-25.23	-0.29	-23.65	-0.27	-46.05	-0.31	-46.12	-0.31	-44.32	-0.29	-66.91	-0.33	-69.41	-0.31	-60.99
-0.32	-28.48	-0.30	-24.53	-0.28	-23.07	-0.28	-45.44	-0.32	-45.91	-0.32	-44.06	-0.30	-66.84	-0.34	-70.29	-0.32	-61.00
-0.31	-27.56	-0.29	-23.95	-0.27	-22.51	-0.29	-45.00	-0.33	-45.76	-0.33	-43.93	-0.31	-66.93	-0.35	-71.11	-0.33	-61.22
-0.30	-26.41	-0.28	-23.42	-0.26	-21.98	-0.30	-44.65	-0.34	-45.73	-0.34	-43.81	-0.32	-67.27	-0.36	-72.12	-0.34	-61.66
-0.29	-25.52	-0.27	-22.90	-0.25	-21.49	-0.31	-44.37	-0.35	-45.79	-0.35	-43.81	-0.33	-67.58	-0.37	-73.33	-0.35	-62.20
-0.28	-24.70	-0.26	-22.67	-0.24	-21.02	-0.32	-44.21	-0.36	-45.92	-0.36	-43.90	-0.34	-68.03	-0.38	-74.56	-0.36	-62.88
-0.27	-23.95	-0.25	-21.94	-0.23	-20.61	-0.33	-44.09	-0.37	-46.17	-0.37	-44.08	-0.35	-68.69	-0.39	-75.86	-0.37	-63.69
-0.26	-23.26	-0.24	-21.50	-0.22	-20.14	-0.34	-44.10	-0.38	-46.50	-0.38	-44.34	-0.36	-69.44	-0.40	-77.15	-0.38	-64.57
-0.25	-22.61	-0.23	-21.08	-0.21	-19.73	-0.35	-44.18	-0.39	-46.92	-0.39	-44.70	-0.37	-70.37	-0.39	-66.48	-0.39	-65.55
-0.24	-22.06	-0.22	-20.67	-0.20	-19.32	-0.36	-44.33	-0.40	-47.40	-0.40	-45.10	-0.38	-71.36	-0.38	-59.22	-0.40	-66.56
-0.23	-21.43	-0.21	-20.28	-0.19	-18.94	-0.37	-44.60	-0.39	-44.59	-0.39	-42.42	-0.39	-72.44	-0.37	-53.58	-0.39	-59.31
-0.22	-20.98	-0.20	-19.90	-0.18	-18.56	-0.38	-44.96	-0.38	-42.43	-0.38	-40.37	-0.40	-73.53	-0.36	-48.90	-0.38	-54.30
-0.21	-20.49	-0.19	-19.53	-0.17	-18.19	-0.39	-45.39	-0.37	-40.60	-0.37	-38.60	-0.39	-64.60	-0.35	-44.98	-0.37	-50.35
-0.20	-19.90	-0.18	-19.17	-0.16	-17.83	-0.40	-45.86	-0.36	-38.96	-0.36	-37.04	-0.38	-58.51	-0.34	-41.57	-0.36	-47.02
-0.19	-19.46	-0.17	-18.83	-0.15	-17.48	-0.39	-42.58	-0.35	-37.52	-0.35	-35.64	-0.37	-53.74	-0.33	-38.55	-0.35	-44.16
-0.18	-18.97	-0.16	-18.48	-0.14	-17.13	-0.38	-40.15	-0.34	-36.18	-0.34	-34.37	-0.36	-49.74	-0.32	-35.92	-0.34	-41.63
-0.17	-18.52	-0.15	-18.14	-0.13	-16.79	-0.37	-38.12	-0.33	-34.95	-0.33	-33.20	-0.35	-46.35	-0.31	-33.51	-0.33	-39.37
-0.16	-18.10	-0.14	-17.81	-0.12	-16.46	-0.36	-36.38	-0.32	-33.82	-0.32	-32.15	-0.34	-43.37	-0.30	-31.37	-0.32	-37.34
-0.15	-17.68	-0.13	-17.49	-0.11	-16.13	-0.35	-34.80	-0.31	-32.79	-0.31	-31.16	-0.33	-40.73	-0.29	-29.44	-0.31	-35.48
-0.14	-17.27	-0.12	-17.17	-0.10	-15.80	-0.34	-33.37	-0.30	-31.84	-0.30	-30.27	-0.32	-38.38	-0.28	-27.74	-0.30	-33.80
-0.13	-16.87	-0.11	-16.85	-0.09	-15.48	-0.33	-32.08	-0.29	-30.97	-0.29	-29.45	-0.31	-36.23	-0.27	-26.18	-0.29	-32.26
-0.12	-16.47	-0.10	-16.54	-0.08	-15.16	-0.32	-30.91	-0.28	-30.18	-0.28	-28.70	-0.30	-34.30	-0.26	-24.78	-0.28	-30.88
-0.11	-16.10	-0.09	-16.23	-0.07	-14.84	-0.31	-29.83	-0.27	-29.44	-0.27	-28.00	-0.29	-32.55	-0.25	-23.50	-0.27	-29.61
-0.10	-15.71	-0.08	-15.92	-0.06	-14.52	-0.30	-28.85	-0.26	-28.77	-0.26	-27.35	-0.28	-30.97	-0.24	-22.31	-0.26	-28.46
-0.09	-15.33	-0.07	-15.61	-0.05	-14.20	-0.29	-27.96	-0.25	-28.14	-0.25	-26.75	-0.27	-29.53	-0.23	-21.26	-0.25	-27.39
-0.08	-14.95	-0.06	-15.30	-0.04	-13.88	-0.28	-27.14	-0.24	-27.55	-0.24	-26.19	-0.26	-28.25	-0.22	-20.28	-0.24	-26.39
-0.07	-14.57	-0.05	-15.00	-0.03	-13.56	-0.27	-26.39	-0.23	-27.01	-0.23	-25.67	-0.25	-27.03	-0.21	-19.39	-0.23	-25.49
-0.06	-14.20	-0.04	-14.69	-0.02	-13.24	-0.26	-25.70	-0.22	-26.50	-0.22	-25.17	-0.24	-25.91	-0.20	-18.55	-0.22	-24.65
-0.05	-13.82	-0.03	-14.38	-0.01	-12.91	-0.25	-25.05	-0.21	-26.01	-0.21	-24.71	-0.23	-24.90	-0.19	-17.84	-0.21	-23.89
-0.04	-13.46	-0.02	-14.07			-0.24	-24.45	-0.20	-25.56	-0.20	-24.26	-0.22	-23.94	-0.18	-17.15	-0.20	-23.14
-0.03	-13.08	-0.01	-13.75			-0.23	-23.90	-0.19	-25.13	-0.19	-23.85	-0.21	-23.09	-0.17	-16.47	-0.19	-22.51
-0.02	-12.70					-0.22	-23.37	-0.18	-24.71	-0.18	-23.45	-0.20	-22.25	-0.16	-15.89	-0.18	-21.89
-0.01	-12.32					-0.21	-22.89	-0.17	-24.31	-0.17	-23.06	-0.19	-21.53	-0.15	-15.32	-0.17	-21.27
						-0.20	-22.41	-0.16	-23.93	-0.16	-22.70	-0.18	-20.82	-0.14	-14.79	-0.16	-20.72
						-0.19	-21.97	-0.15	-23.56	-0.15	-22.33	-0.17	-20.14	-0.13	-14.28	-0.15	-20.20
						-0.18	-21.56	-0.14	-23.20	-0.14	-21.97	-0.16	-19.53	-0.12	-13.82	-0.14	-19.69
						-0.17	-21.15	-0.13	-22.83	-0.13	-21.63	-0.15	-18.94	-0.11	-13.41	-0.13	-19.21
						-0.16	-20.77	-0.12	-22.49	-0.12	-21.30	-0.14	-18.37	-0.10	-12.97	-0.12	-18.77

						-0.15	-20.40	-0.11	-22.17	-0.11	-20.98	-0.13	-17.84	-0.09	-12.56	-0.11	-18.37
						-0.14	-20.03	-0.10	-21.84	-0.10	-20.66	-0.12	-17.34	-0.08	-12.19	-0.10	-17.95
						-0.13	-19.68	-0.09	-21.52	-0.09	-20.35	-0.11	-16.89	-0.07	-11.84	-0.09	-17.54
						-0.12	-19.35	-0.08	-21.21	-0.08	-20.04	-0.10	-16.42	-0.06	-11.48	-0.08	-17.16
						-0.11	-19.03	-0.07	-20.90	-0.07	-19.75	-0.09	-15.97	-0.05	-11.15	-0.07	-16.80
						-0.10	-18.70	-0.06	-20.59	-0.06	-19.45	-0.08	-15.55	-0.04	-10.83	-0.06	-16.43
						-0.09	-18.38	-0.05	-20.30	-0.05	-19.16	-0.07	-15.16	-0.03	-10.54	-0.05	-16.08
						-0.08	-18.07	-0.04	-20.00	-0.04	-18.88	-0.06	-14.75	-0.02	-10.23	-0.04	-15.74
						-0.07	-17.78	-0.03	-19.72	-0.03	-18.59	-0.05	-14.37	-0.01	-9.92	-0.03	-15.42
						-0.06	-17.48	-0.02	-19.42	-0.02	-18.31	-0.04	-14.00			-0.02	-15.07
						-0.05	-17.19	-0.01	-19.13	-0.01	-18.02	-0.03	-13.65			-0.01	-14.74
						-0.04	-16.89					-0.02	-13.30				
						-0.03	-16.61					-0.01	-12.94				
						-0.02	-16.32										
						-0.01	-16.03										

The triplicates for each condition are represented by the numbers 1 to 3.

Supplementary Table 2. Results of variant effect prediction algorithms

Non-pathogenic Variant	Polyphen-2[1]	SNAP-2[2]	Provean[3]	Hope
p.(Ala444Thr)	0,992	33	-3,539	bigger, negative>neutral, more hydrophobic
p.(Asp418Gly)	1	89	-6,733	smaller, negative>neutral, more hydrophobic
p.(Glu369Gly)	0,763	56	-3,641	smaller, negative>neutral, more hydrophobic
p.(Phe396Cys)	0,995	36	-4,975	smaller
p.(Ser419Asn)	0,866	53	-2,88	bigger, more hydrophobic
p.(Tyr319Asp)	0,999	82	-8,141	smaller, more hydrophobic
p.(Tyr319Cys)	0,998	53	-7,574	smaller, negative>neutral, more hydrophobic

[1] The results range from zero to one, in that scenario zero is considered benign and one damaging.

[2] Higher and positive results indicates higher effects in protein functions.

[3] Scores below to -2.5 are considered deleterious while above are considered neutral.
